# Secretion of the siderophore rhizoferrin is regulated by the cAMP-PKA pathway and is involved in the virulence of *Mucor lusitanicus*

**DOI:** 10.1038/s41598-022-14515-0

**Published:** 2022-06-23

**Authors:** Viridiana Alejandre-Castañeda, J. Alberto Patiño-Medina, Marco I. Valle-Maldonado, Rosa E. Nuñez-Anita, Gustavo Santoyo, Karla V. Castro-Cerritos, Rafael Ortiz-Alvarado, Alma R. Corrales-Escobosa, Martha I. Ramírez-Díaz, J. Felix Gutiérrez-Corona, Adolfo López-Torres, Victoriano Garre, Víctor Meza-Carmen

**Affiliations:** 1 Instituto de Investigaciones Químico Biológicas, Universidad Michoacana de San Nicolás de Hidalgo (UMSNH), Morelia, Michoacán Mexico; 2 Departamento de Biología Molecular, Laboratorio Estatal de Salud Pública del Estado de Michoacán, Morelia, Michoacán Mexico; 3 Facultad de Medicina Veterinaria y Zootecnia, Universidad Michoacana de San Nicolás Hidalgo, Morelia, Michoacán Mexico; 4 Instituto de Química Aplicada, Universidad Papaloapan, Campus Tuxtepec, Tuxtepec, Oaxaca, Mexico; 5 Facultad de Químico Farmacobiología, Universidad Michoacana de San Nicolás de Hidalgo, Morelia, Michoacán Mexico; 6 Departamento de Química, División de Ciencias Naturales y Exactas, Universidad de Guanajuato, Guanajuato, Guanajuato Mexico; 7 Departamento de Biología, División de Ciencias Naturales y Exactas, Universidad de Guanajuato, Guanajuato, Guanajuato Mexico; 8 Departamento de Genómica y Biotecnología Molecular de Hongos, Universidad de Murcia, Murcia, Spain; 9 Laboratorio de Diferenciación Celular, Instituto de Investigaciones Químico Biológicas, Universidad Michoacana de San Nicolás de Hidalgo, Ciudad Universitaria 58030, Morelia, Michoacán Mexico

**Keywords:** Microbiology, Molecular biology

## Abstract

Mucormycosis is a fungal infection caused by Mucorales, with a high mortality rate. However, only a few virulence factors have been described in these organisms. This study showed that deletion of *rfs*, which encodes the enzyme for the biosynthesis of rhizoferrin, a siderophore, in *Mucor lusitanicus*, led to a lower virulence in diabetic mice and nematodes. Upregulation of *rfs* correlated with the increased toxicity of the cell-free supernatants of the culture broth (SS) obtained under growing conditions that favor oxidative metabolism, such as low glucose levels or the presence of H_2_O_2_ in the culture, suggesting that oxidative metabolism enhances virulence through rhizoferrin production. Meanwhile, growing *M. lusitanicus* in the presence of potassium cyanide, N-acetylcysteine, a higher concentration of glucose, or exogenous cAMP, or the deletion of the gene encoding the regulatory subunit of PKA (*pkaR1*), correlated with a decrease in the toxicity of SS, downregulation of *rfs*, and reduction in rhizoferrin production. These observations indicate the involvement of the cAMP-PKA pathway in the regulation of rhizoferrin production and virulence in *M. lusitanicus*. Moreover, *rfs* upregulation was observed upon macrophage interaction or during infection with spores in mice, suggesting a pivotal role of *rfs* in *M. lusitanicus* infection.

## Introduction

*Mucor lusitanicus* (formerly *M. circinelloides* f. *lusitanicus*)^1^ is a dimorphic fungus belonging to the sub-phylum Mucormycotina^2^. It is a biological model for mucormycosis, a rare but fatal infection^3–5^. Iron has a critical function in oxidative metabolism as it promotes adequate mitochondrial and antioxidant system function^6,7^. Three principal mechanisms of iron acquisition are reported in pathogenic fungi: reductive iron uptake, siderophore synthesis, and transport^8^^.^ To cause infection, Mucorales must acquire iron from the host for successful growth^9^. The ferroxidases Fet3a, Fet3b, and Fet3c are expressed at high levels in *M. lusitanicus* during infections in mice compared to that in in vitro culture and are critical factors for determining the full virulent phenotype^10^. In *Rhizopus oryzae*, the high-affinity iron permease Ftr1 positively regulates iron acquisition during growth and virulence during infection in mice^11,12^. Moreover, several Mucorales causing mucormycosis, including *M. circinelloides,* secrete a siderophore named rhizoferrin, which belongs to the family of polycarboxylates and is synthetized by a non-ribosomal peptide synthetase NRPS-independent siderophore enzyme encoded by *rfs*^13^. Rhizoferrin could be involved in conferring virulence in Mucorales, but this hypothesis remains to be verified^14^.

In addition to the levels of iron available, dimorphism plays an important role in virulence. The ability to grow as mycelium or yeast depends primarily on the concentration of oxygen or carbon in the culture of *M. lusitanicus*^15,16^. Mycelial or yeast growth generally results in an increase in oxidative or fermentative metabolism, respectively^17,18^. Moreover, hyphal growth corresponds to greater virulence than yeast growth^19,20^.

The regulation of virulence in *M. lusitanicus* involves several factors that affect mycelial growth, such as calcineurin, heterotrimeric G-proteins, cAMP-PKA, and ADP-ribosylation factors (Arf), which are vesicle regulators, and an imbalance in fermentative-oxidative metabolism*.* Mutations in *cnbR*, which encodes the regulatory subunit of calcineurin, led to a monomorphic yeast-locked phenotype, even during aerobic growth and decreased virulence^19^. Meanwhile, the catalytic calcineurin CnaA subunit is involved in the negative regulation of virulence^21^. The cAMP-PKA pathway positively regulates mycelial growth and virulence; mutations in *pkaR1*, as well as the deletion of *gpb1*, which encodes the heterotrimeric G-beta subunit 1, resulted in decreased virulence and increased yeast growth under low oxygen levels^22^. Arf3 and Arl1 are involved in the regulation of secreted virulence factors^23,24^ and positively control the mitochondrial content during aerobic growth^25^. However, information regarding the involvement of molecules excreted/secreted by *M. lusitanicus* in virulence is limited. Increased excretion of acetaldehydes owing to mutations in *adh1* (encoding alcohol dehydrogenase 1) is associated with increased *M. lusitanicus* virulence^20^, and the secreted toxin mucoricin plays an important role in Mucorales pathogenesis^26^.

Here, we analyzed the physiological, molecular, and virulence-related implications of an Rfs-encoding enzyme and its product rhizoferrin. We used a physiological and loss-of-function approach to describe the role of rhizoferrin in the virulence of *M. lusitanicus*.

## Results

### Culture broth composition influences the virulence of *M. lusitanicus* during mycelial growth

**Figure 1:**
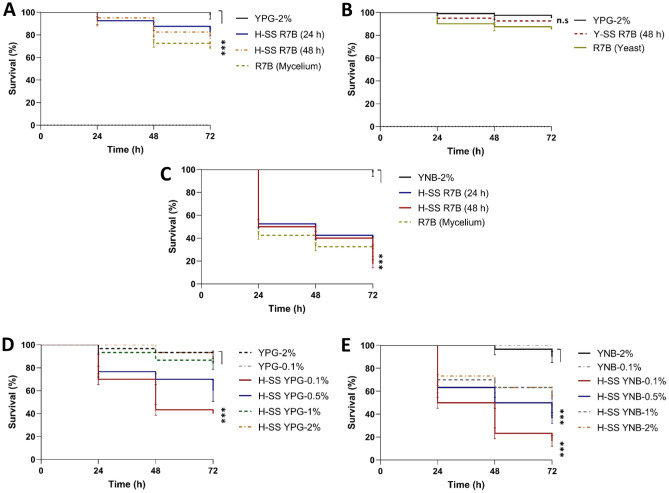
Analysis of the toxicity of the cell-free supernatant of the culture broth (SS) of *M. lusitanicus* under different growth conditions. Quantification of the toxicity of the SS obtained from the wild-type strain R7B grown in YPG medium with 2% glucose (YPG-2%) after (**A**) aerobic growth (H-SS) or (**B**) anaerobic growth (Y-SS) at indicated times against *Caenorhabditis elegans*; hyphae and yeast cells obtained from 3 and 8 h, respectively, were recovered under the corresponding growth conditions and tested against the nematode. (**C**) Hyphae from a culture with 3 h of growth or its SS obtained from the YNB medium with 2% glucose (YNB-2%) after aerobic growth at indicated times were used to treat the nematode. Quantification of the toxicity of the SS obtained after aerobic growth for 48 h in (**D**) YPG medium and (**E**) YNB medium supplemented with different concentrations glucose (0.1, 0.5, 1, and 2%). A total of 15–20 nematodes were used per experiment and incubated at 18 °C for 72 h. The results presented are the average values obtained from four independent experiments. Data were statistically analyzed using the Mantel-Cox test. ***P < 0.01. When results were not considered significant, we did not provide an additional indication (*P *> 0.05).

To examine the correlation between the growth conditions and virulence of *M. lusitanicus*, vegetative cells and cell-free supernatants of the culture broth (SS) obtained after the growth of the wild-type (WT) strain R7B were evaluated under different growth conditions. Cultures of mycelium or yeast and their corresponding SSs obtained in standard YPG medium containing 2% glucose (YPG-2%) were assayed against a culture of the nematode *Caenorhabditis elegans*. The mycelium and their SSs exhibited a marginally higher virulence than the yeast or its corresponding SSs (Fig. 1A,B). Therefore, mycelial SS was used in this study. The presence of an inorganic nitrogen source, such as ammonium sulfate present in the YNB medium, was evaluated. The SSs from R7B grown in standard YNB medium supplemented with 2% glucose (YNB-2%) or mycelium growing in YNB-2% led to a death rate of 60%–70% in the nematodes (Fig. 1C), which was higher than that caused by the mycelium and their SSs from YPG-2% (Fig. 1A).

The mRNA levels of *pkaR1* and *atp9* (mitochondrial gene that encodes a subunit of the mitochondrial ATPase), the increase in which correlated with mycelial growth and mitochondrial activity, respectively^27,28^, were higher (≈ 66% for *pkaR1* and ≈ 24% for *atp9*) in the mycelium obtained from YNB-2% after 8 h of growth than in that obtained from YPG-2% (Fig. S1A). The *adh1* mRNA level, which indirectly indicates the fermentative state^18^, was slightly lower in the mycelium grown in YNB-2% than in that grown in YPG-2% (Fig. S1A). Therefore, the inorganic nitrogen source increased oxidative metabolism and the virulence of *M. lusitanicus*. Corroborating this hypothesis, the addition of peptone (at the same concentration as that in YPG) to YNB-2% reduced the toxicity of the SSs to levels similar to those observed in SSs obtained from YPG-2% (Fig. 1A and Fig. S1B).

Moreover, decreasing the glucose concentration to less than 2% in the YPG or YNB media increased the toxicity of the SSs from R7B (Fig. 1D,E). Additionally, the SS from mycelium grown in YNB supplemented with glycerol as a non-fermentable carbon source also exhibited a high toxicity against the nematode (Fig. S1B). The toxicity of SS obtained from the mycelium of the MU636 and MU402 strains was also examined by either lowering the glucose concentration or adding ammonium sulfate in the media, which indicated that these conditions enhance the toxicity of the SSs (Fig. S2). In general, these results indicated the influence of the culture condition on the modulation of the virulence of *M. lusitanicus.*

### Toxic factors are sensitive to protease activity and have a low molecular weight (MW)

**Figure 2:**
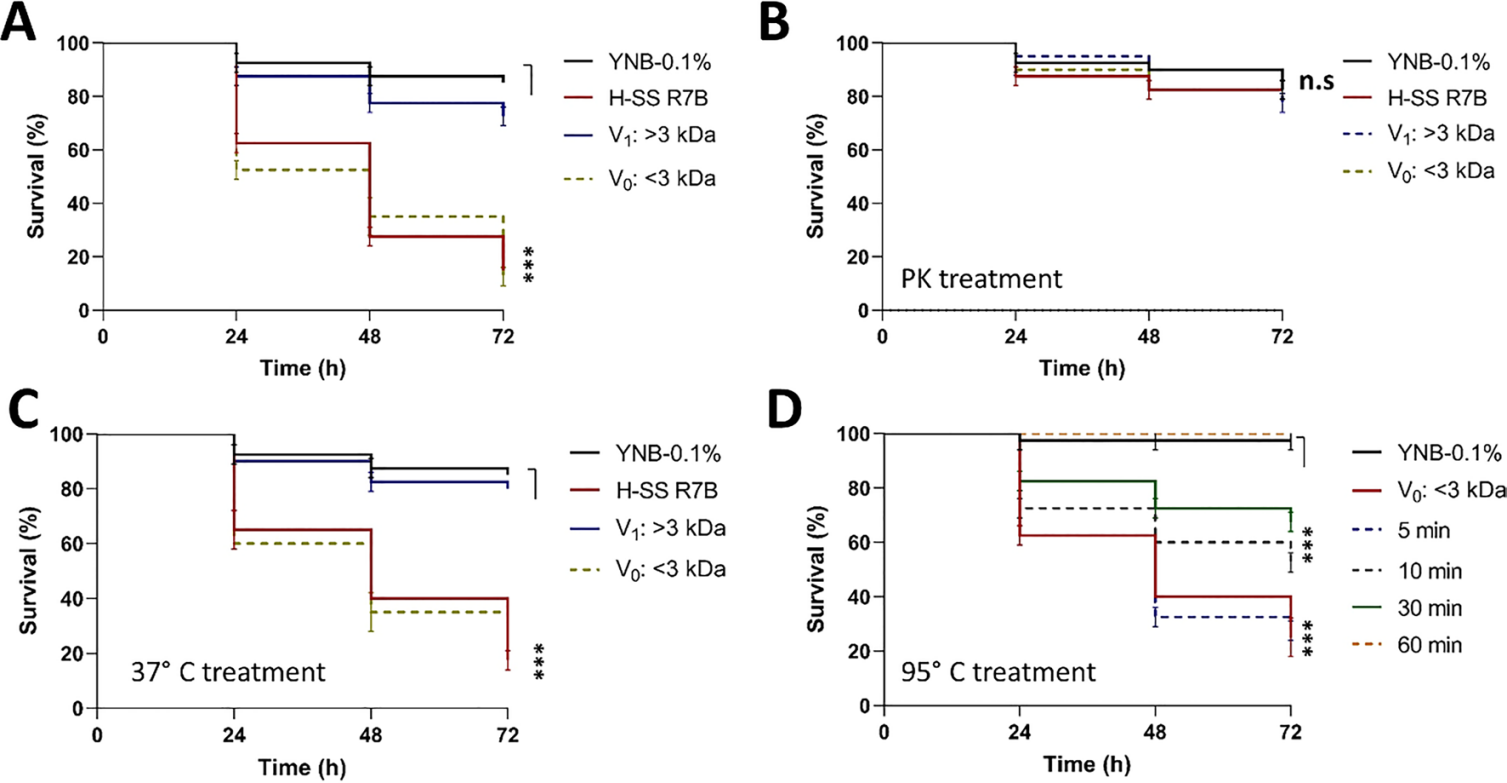
The toxic factor in the cell-free supernatant of the culture (SS) of *M. lusitanicus* was susceptible to protease activity and had a low molecular weight. (**A**) SS from the R7B strain obtained under aerobic conditions (H-SS) in YNB medium supplemented with 0.1% glucose (YNB-0.1%) after 48 h of growth was passed through a molecular weight exclusion filter (cut off 3 kDa), obtaining a V_o_ fraction consisting of molecules with a weight lesser than 3 kDa and a V_1_ fraction consisting of molecules with a weight greater than 3 kDa. (**B**) SS or V_o_ and V_1_ fractions from panel A were treated with proteinase K (PK) for 2 h at 37 °C; SS or V_o_ and V_1_ fractions from panel A were incubated for 2 h at (**C**) 37 °C or (**D**) 95 °C. All the SSs and fractions were used to treat *Caenorhabditis elegans* to test their toxicity. A total of 15–20 nematodes were used per experiment and incubated at 18 °C for 72 h. The results presented are the average of the values obtained from four independent experiments. The data were statistically analyzed using the Mantel-Cox test. ***P < 0.01. When results were considered not significant, we did not provide an additional indication (*P *> 0.05).

To determine the MW and chemical nature of the toxic factors, the SS obtained from the R7B strain grown for 48 h in the YNB medium with 0.1% glucose (YNB-0.1%) was separated using a MW exclusion membrane with a cut-off of 3 kDa. Two fractions were obtained: V_0_ corresponding to molecules with a MW < 3 kDa, and V_1_ corresponding to molecules with MW > 3 kDa. The SS and V_0_ fraction induced a similar rate of nematode death (≈ 85%) (Fig. 2A). In contrast, the V_1_ fraction induced a rate of death (< 20%) similar to that of the control. The incubation of SS or the V_0_ fraction with proteinase K (PK) for 2 h at 37 °C inhibited the toxicity of both samples (Fig. 2B). Meanwhile, the incubation of the SS or V_0_ fraction under the same conditions without PK exerted no effect (Fig. 2C). Both the SS and V_0_ fraction maintained their full toxicity after 5 min of incubation at 95 °C, but the toxicity was reduced to 45% after 10 min or inhibited after 60 min of incubation at a high temperature (Fig. 2D). These results indicate that the molecules in the SS from R7B that exhibited toxicity had a MW lesser than 3 kDa, were susceptible to protease activity, and exhibited complete thermic resistance for 5 min at 95 °C.

### Toxicity of the cell-free supernatants of the culture broths is repressed by the presence of Fe^2+^

The low MW and protease sensitivity of the toxic factors present in the SS from *M. lusitanicus* suggested that the toxic factor could be the product of the non-ribosomal peptide synthetase, Rfs, which synthesizes the siderophore rhizoferrin produced by several Mucorales^13^ and has a MW of 436.37 Da^29^.

**Figure 3:**
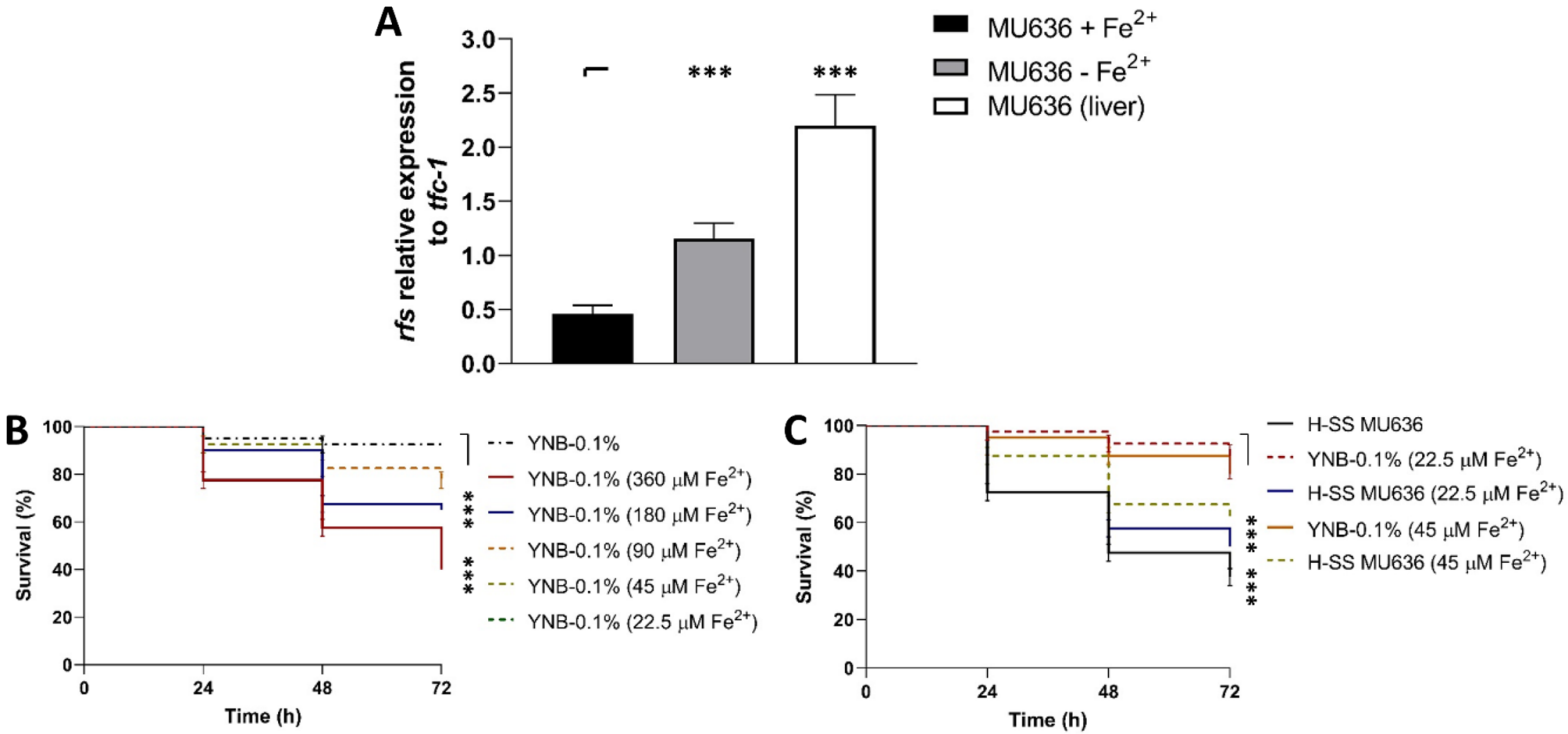
Addition of Fe^2+^ downregulates *rfs* expression and negatively influences the virulence of the cell-free supernatant of the culture (SS) from *M. lusitanicus*. (**A**) Quantification of the mRNA levels of *rfs* in the mycelium of MU636 grown for 48 h under aerobic conditions in Vogel´s medium supplemented with 2% glucose with or without supplementation with 200 μM Fe^2+^ or in the livers of diabetic mice infected previously with 3 × 10^6^ spores of the MU636 strain and euthanized 7 days post infection; a ∆Ct analysis was performed to compare the mRNA levels between samples. *tfc1* was used as reference housekeeping gene (**B**) Effect of Fe^2+^ present at different concentrations in the YNB medium with 0.1% glucose (YNB-0.1%) on nematode survival. (**C**) Toxicity of the SSs obtained when the fungus (strain MU636) was aerobically grown (H-SS) for 48 h in YNB-0.1% supplemented with 22.5 μM or 45 μM Fe^2+^. All the SSs were used to treat *Caenorhabditis elegans*. A total of 15–20 nematodes were used per well and incubated at 18 °C for 72 h. Significance testing was performed using ANOVA with Fisher's exact test, *P < 0.05; **P < 0.01; ***P < 0.001. The virulence results presented are the average from four independent experiments. Data were statistically analyzed using the Mantel-Cox test. ***P < 0.01. When results were not considered significant, we did not provide an additional indication (*P* > 0.05).

The synthesis of siderophores is influenced by the absence or presence of iron^30^. The mRNA levels of *rfs* were higher (2.5-fold) in the mycelium grown in Vogel´s medium non-supplemented with Fe^+2^ than in that grown in the presence of 200 μM Fe^2+^ (Fig. 3A). Additionally, the livers of diabetic mice previously infected intraperitoneally with spores from MU636 showed higher mRNA levels of *rfs* than did mycelia grown in the presence or absence of iron (Fig. 3A).

Next, the survival of the nematodes was tested in YNB-0.1% supplemented with different concentrations of Fe^2+^ (22.5–360 μM), revealing that incubation with 22.5 μM and 45 μM of Fe^2+^ led to similar patterns of nematode survival as that observed upon incubation under excess Fe^2+^ supplementation (Fig. 3B). The SSs obtained after the growth of strain MU636 for 48 h in YNB-0.1% supplemented with 22.5 μM and 45 μM Fe^2+^ induced fewer nematode death than did the SSs from YNB-0.1% not supplemented with Fe^+2^ (Fig. 3C). These data indicate that the toxicity of SS of *M. lusitanicus* is suppressed by Fe^2+^ present in the culture medium. Furthermore, the mRNA levels of *rfs* were upregulated by low levels of Fe^2+^ or by the infection process in mice.

### Deletion of *rfs* decreased the toxicity of the cell-free supernatants of the culture broth

To determine the role of Rfs in the toxicity of the SS from *M. lusitanicus*, a recombinant fragment containing *pyrG* flanked by a ≈1-kb fragment corresponding to the 5ʹ upstream site of ATG and 3ʹ downstream site of the stop codon of *rfs* was used to delete the *rfs* in MU402 (*pyrG*^*−*^, *leuA*^*−*^) (Fig. S3A). The deletion was confirmed via PCR using specific primers, revealing the presence of the recombinant fragment in the *rfs* locus in the mutant strains, indicating a homokaryotic genotype (Fig. S3B and S3C) or the integrity of the *rfs* gene in the genome of the wild-type strain (WT) (Fig. S3C). Characterization of two independent *rfs* mutant strains (Fig. S3C) was performed with similar results, and we only included the results from one (∆*rfs-1* named as ∆*rfs* in the whole manuscript, Fig. S3C) of these strains.

**Figure 4:**
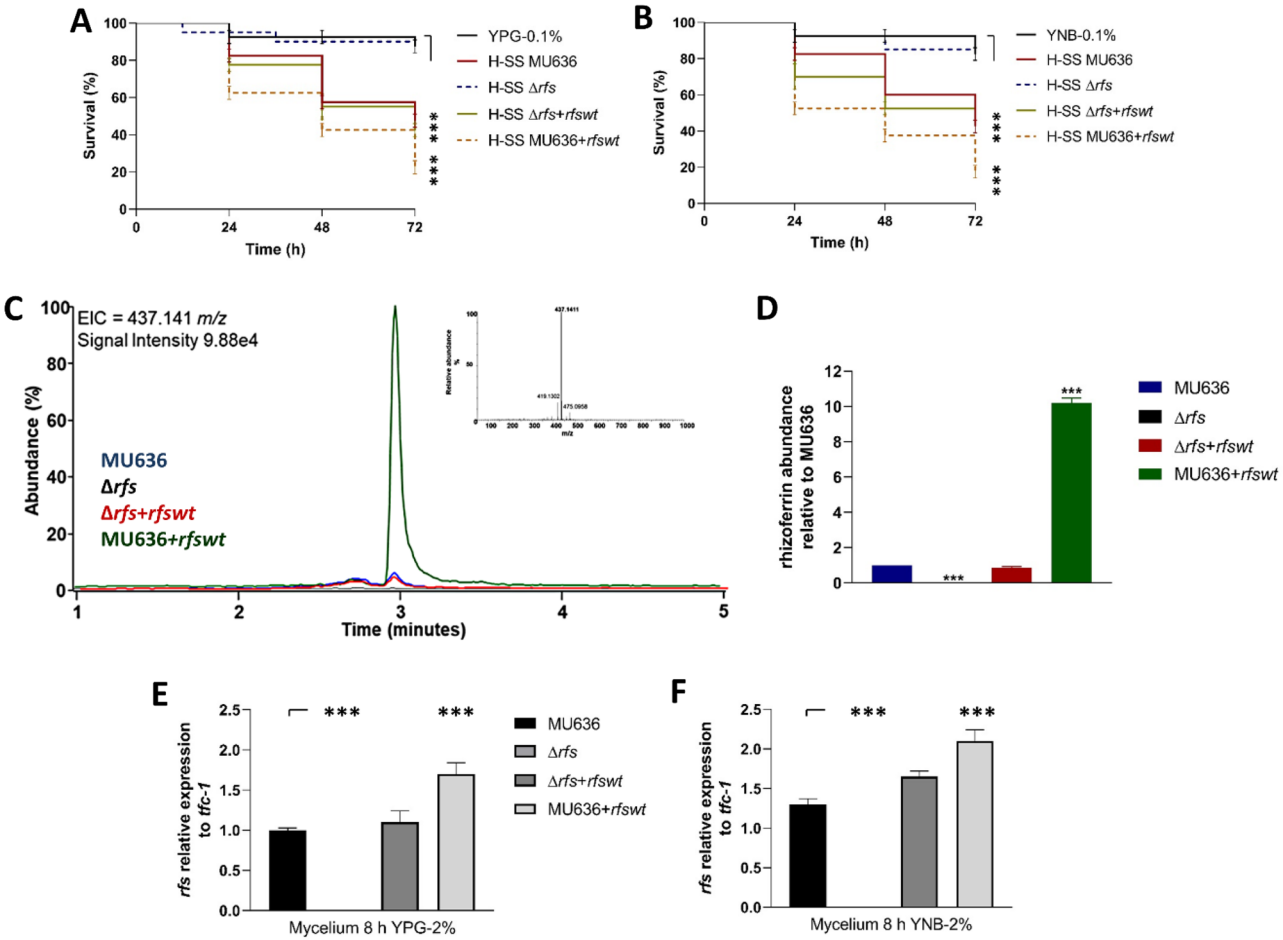
*rfs* is essential for the toxicity of the cell-free supernatant of the culture (SS) of *M. lusitanicus*. The toxicity of the SSs obtained from the different strains after 48 h of aerobic growth (H-SS) in (**A**) YPG medium (YPG-0.1%) and (**B**) YNB medium (YNB-0.1%) supplemented with 0.1% glucose was assessed against the nematode. A total of 15–20 nematodes were used per experiment and incubated at 18 °C for 72 h. The results presented are the average of results obtained from four independent experiments. Data were statistically analyzed using the Mantel-Cox test. ***P < 0.01. When results were not considered significant, we did not provide an additional indication (*P* < 0.1). (**C**) Extracted ion chromatograms of rhizoferrin (*m/z* = 437.141) in SS obtained from the different *M. lusitanicus* strains after 48 h of growth in the YNB medium with 0.1% glucose. Chromatograms show the relative abundance of the most intense sample. Top, a representative mass spectrum of rhizoferrin. (**D**) Relative abundance of rhizoferrin in the corresponding SS from the different strains. Total RNA was obtained from mycelium grown for 8 h either in (**E**) YPG medium (with 2% glucose) or (**E**) YNB medium (with 2% glucose), and *rfs* expression was assayed by RT-qPCR. A ∆Ct analysis was performed to compare the mRNA levels between samples, using *tfc1* as reference housekeeping gene. Significance testing was performed using ANOVA with Fisher's exact test, *P < 0.05; **P < 0.01; ***P < 0.001.

The corresponding SSs obtained after 48 h from the mycelia of MU636 (WT), ∆*rfs* (mutant), ∆*rfs* + *rfswt* (*rfs*-complemented), and MU636 + *rfswt* (*rfs-*overexpressing) grown in YPG-0.1% or YNB-0.1% were used in virulence assays against nematodes, revealing that the SS from ∆*rfs* led to an increase in nematode survival, in contrast to those of the rest of the strains that caused greater death in nematodes, especially the SS from MU636 + *rfswt* (Fig. 4A,B). Similar results were obtained using spores for the virulence assays (Fig. S4). The decrease in the virulence of ∆*rfs* was not owing to lower levels of growth, because the biomass generated by ∆*rfs* and WT strains after 48 h of growth in YPG was similar, despite its slower germination (Fig. S5). This could be explained by the presence of iron (20 μM) in YPG media, as it has been reported^31^ that this could positively influence ∆*rfs* growth.

The relative quantification of rhizoferrin in the SS obtained from the mycelia of the different strains grown in YNB-0.1% was performed using ultra-performance liquid chromatography coupled with electrospray ionization quadrupole time-of-flight mass spectrometry (UPLC-ESI-TOF–MS). The SS obtained from MU636 showed similar levels of rhizoferrin accumulation as that obtained from Δ*rfs* + *rfswt*, whereas the SS from MU636 + *rfswt* showed 10.2 times more rhizoferrin accumulation than the SS from WT (Fig. 4C,D), and ∆*rfs* showed no rhizoferrin accumulation (Fig. 4C,D).

The mycelium from the strains grown for 8 h in YPG-2% and YNB-2% was used to quantify the mRNA levels of *rfs*. The transcript levels in MU636 + *rfswt* in both media were ≈ 62%–70% higher than those in WT or Δ*rfs* + *rfswt*, whereas no transcript was detected in ∆*rfs*. The mycelium from strains carrying the functional *rfs* gene grown in YNB-2% contained ≈24%–50% more mRNA than those from strains grown in YPG-2% (Fig. 4E,F). In general, these results demonstrate the pivotal role of *rfs* of *M. lusitanicus* in iron homeostasis and toxicity of the cell-free supernatants of the culture broth against nematodes.

### Rfs is required for full virulence in mice

**Figure 5:**
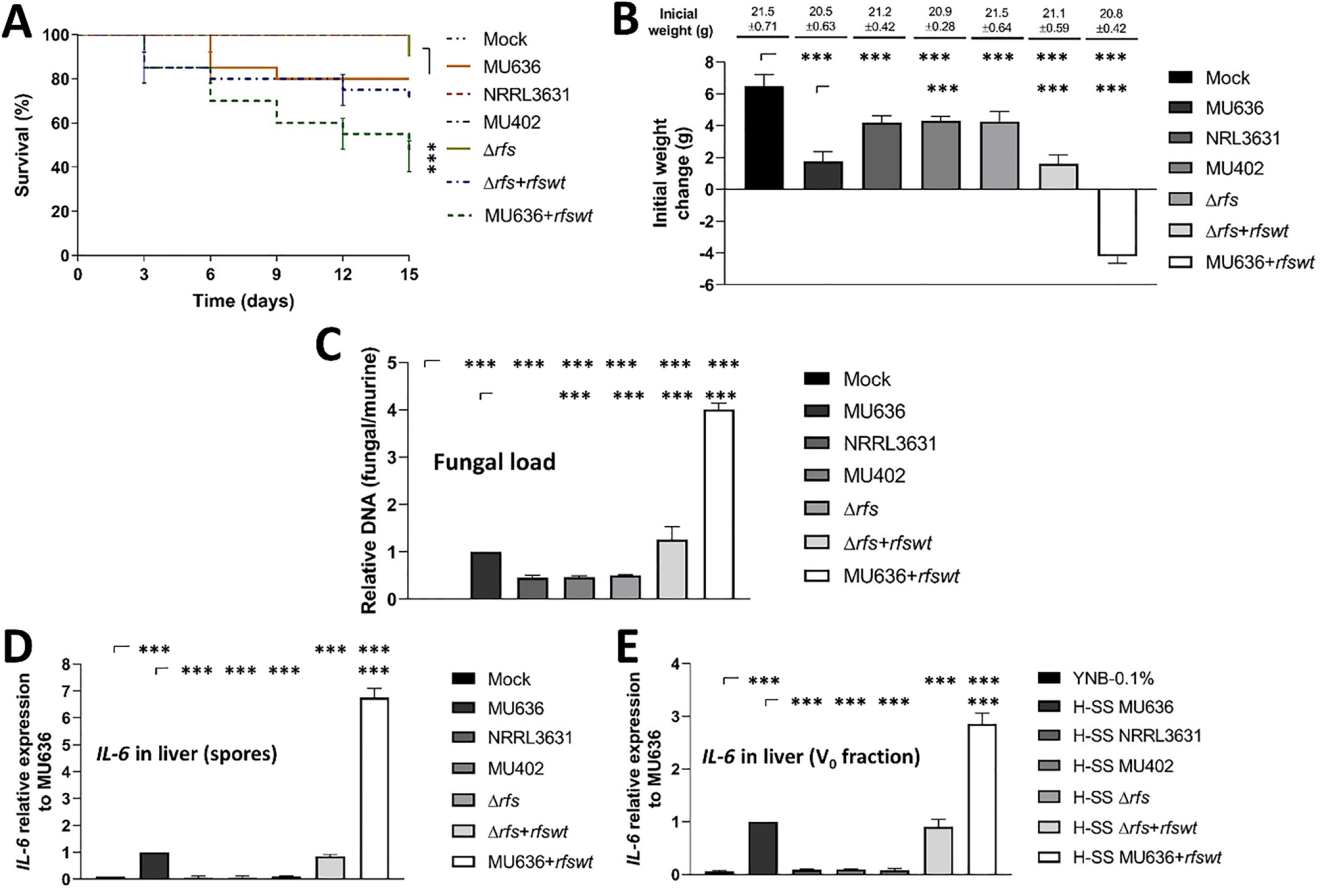
Role of *rfs* in the regulation of *M. lusitanicus* virulence in mice. (**A**) Spores (3 × 10^7^) from the MU636, Δ*rfs*, Δ*rfs* + *rfsw*t, and MU636 + *rfsw*t strains were intraperitoneally injected into streptozotocin-induced diabetic mice, and survival was monitored daily for 15 days. The experiments were repeated two times in independent groups with n = 10. Data were statistically analyzed using the Mantel-Cox test. ***P < 0.01. When results were not considered significant, we did not provide an additional indication (*P* > 0.05). Mice surviving after 7 days post-inoculation were considered to (**B**) determine the change in the weight of mice since the initial infection. Animals were euthanized at 15 days post-inoculation, and the livers were removed to isolate nucleic acids to determine the (**C**) fungal burden in the mice using quantitative polymerase chain reaction (qPCR) for the expression of the *tfc-1* gene from *M. lusitanicus* and *Actb* from *Mus musculus*. A ΔCt analysis was performed to compare the relative abundance of the fungal DNA compared with that of the murine DNA. The mRNA levels of the inflammation marker IL-6 in the liver of mice (**D**) infected with spores from the different strains and (**E**) inoculated with 200 μL of the V_o_ fraction obtained through a molecular weight exclusion filter (cut off 3 kDa) were measured using reverse transcription quantitative polymerase chain reaction (RT-qPCR). Data are presented as the average values obtained from four independent biological replicates; ± corresponds to standard error (SE). Significance testing was performed using ANOVA with Fisher's exact test, *P < 0.05; **P < 0.01; ***P < 0.001.

The intraperitoneal injection of diabetic BALB/c mice with spores from the different strains showed that MU636 + *rfswt* exhibited 60% lethality at 15 days post-inoculation, the WT and Δ*rfs* + *rfswt* exhibited 25% and 30% lethality, respectively, ∆*rfs* caused less than 10% lethality in mice, and the NRRL3631 strain was avirulent in this assay (Fig. 5A), probably because of its delayed germination and higher sensitivity of the cell wall to detergents, as described before^32^. Animals infected with MU636 + *rfswt* showed the highest loss of weight (≈ 20%) at 7 days post-infection. Animals infected with ∆*rfs* exhibited a similar increment in weight as the avirulent strains, followed by those infected with the WT or Δ*rfs* + *rfswt* (Fig. 5B). The livers of mice infected with Δ*rfs* as well as MU402 or NRRL3631 exhibited lower fungal loads than the tissues of animals infected with WT or Δ*rfs* + *rfswt* (Fig. 5C). Meanwhile, tissues from animals infected with MU636 + *rfswt* exhibited the highest fungal loads (Fig. 5C).

The livers of mice infected with spores from MU636 + *rfswt* or its V_o_ fraction (obtained similarly as that in Fig. 2) showed the highest mRNA levels of the inflammation marker *IL-6* compared to those of animals infected with spores or their V_o_ fractions from MU636 and Δ*rfs* + *rfswt*, whereas Δ*rfs* induced the lowest levels of mRNA expression of this inflammatory marker (Fig. 5D,E). These results indicated that the deletion of *rfs* decreased the virulence in diabetic mice and the overexpression of *rfs* increased the virulence.

### Macrophage interaction and oxidative stress increased the mRNA levels of *rfs* and production of rhizoferrin

**Figure 6:**
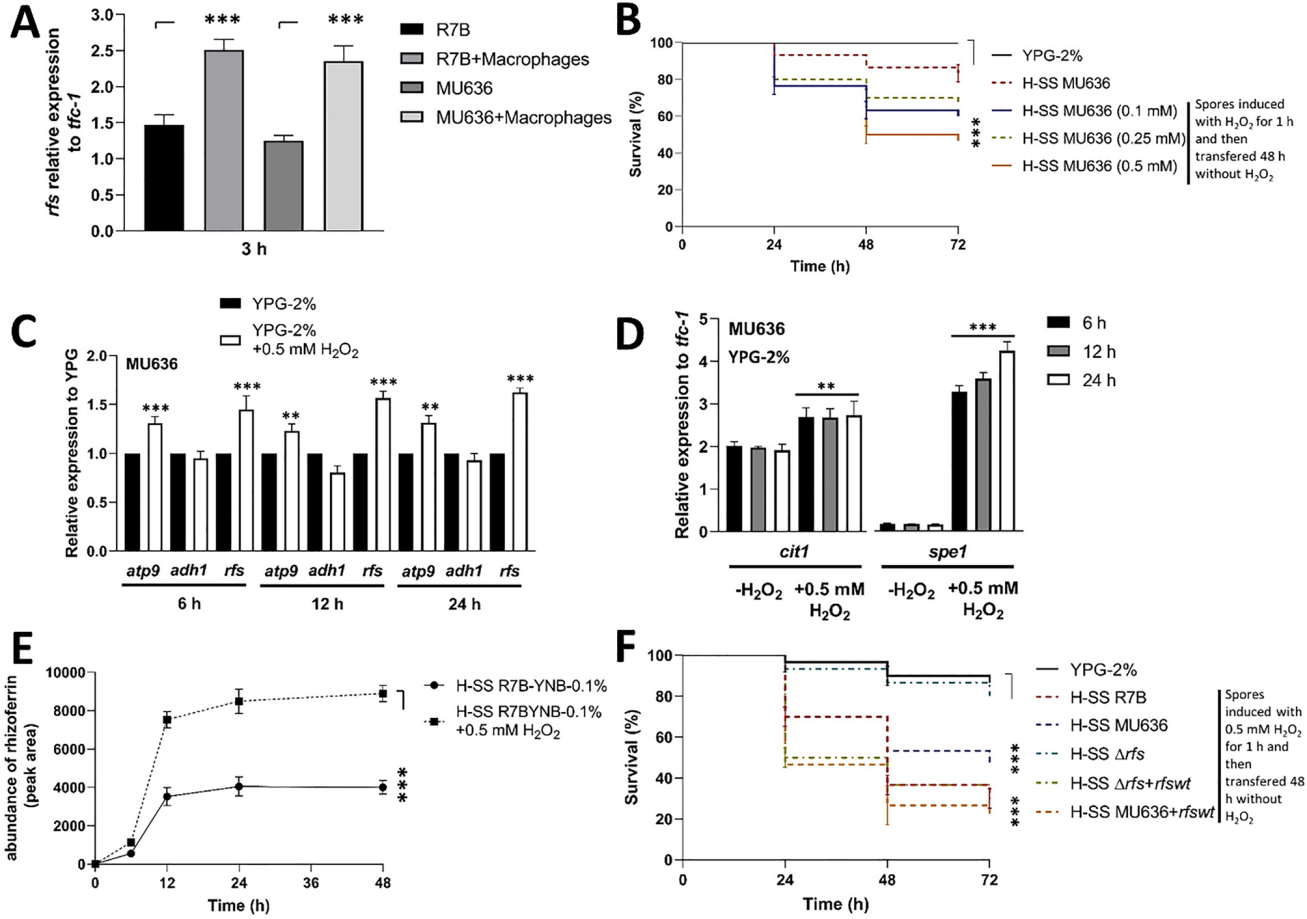
Virulence, *rfs* transcript levels, and rhizoferrin accumulation are influenced by oxidative stress and macrophage interaction. (**A**) mRNA levels of *rfs* in *Mucor lusitanicus* were determined after interaction with macrophages (or not) for 6 h. (**B**) Spores of the MU636 strain were incubated with different concentrations of H_2_O_2_ for 1 h in YPG with 2% glucose (YPG-2%), following which the germinating spores were grown aerobically for 48 h in YPG-2% The toxicity of the cell-free supernatant of the culture (SS) obtained after 48 h of aerobic growth (H-SS) was assessed against the nematode. (**C**) mRNA levels of *atp9*, *adh1,* and *rfs;* (**D**) *cit1* and *spe1* expression was quantified in the mycelium of the MU636 strain grown for 8 h in YPG-2% in the absence or presence of 0.5 mM H_2_O_2_. (**E**) Relative abundance of rhizoferrin in SS obtained from the R7B strain of *M. lusitanicus* grown in YNB with 0.1% glucose (YNB-0.1%) in the absence or presence of 0.5 mM H_2_O_2_. (**F**) The virulence of the SSs obtained after 48 h of growth of different strains in YPG-2% in the absence or presence of 0.5 mM H_2_O_2_ was assessed in the nematode. The SSs were used to treat *Caenorhabditis elegans*. A total of 15–20 nematodes were used per experiment and incubated at 18 °C for 72 h. The results presented are the average of values obtained from four independent experiments. Data were statistically analyzed using the Mantel-Cox test for virulence assays. ***P < 0.01. Significance testing was performed using ANOVA with Fisher's exact test, *P < 0.05; **P < 0.01; ***P < 0.001. When results were not considered significant, we did not provide an additional indication (*P* > 0.05).

After interaction with the mouse RAW267.4 monocytes/macrophage cell line for 3 h, both WT (R7B and MU636) strains showed higher levels of *rfs* mRNA production (71%–88%) than strains without macrophage interaction (Fig. 6A). To determine the effect of oxidative stress on the virulence, MU636 was incubated in YPG-2% in the presence of different concentrations of H_2_O_2_ for 1 h, following which the cells were washed and transferred to fresh YPG-2% for 48 h (without H_2_O_2_), and finally, the SS was obtained to evaluate its toxicity in nematodes. In contrast to the SS obtained from the H_2_O_2_-untreated culture, that obtained after incubation with the peroxide showed greater toxicity at all concentrations of H_2_O_2_ tested (Fig. 6B). The *rfs* mRNA levels were higher (≈ 50%) in mycelium grown under H_2_O_2_-induced oxidative stress than in cultures without H_2_O_2_ (Fig. 6C). Further, the mRNA levels of the mitochondrial marker *atp9* increased by ≈38% in the presence of H_2_O_2_; in contrast, the transcript levels of *adh1*, which indirectly reflects the fermentation state, did not change under this treatment (Fig. 6C). Oxidative stress also positively influenced the accumulation of the transcripts of citrate synthase (*cit1*) and ornithine decarboxylase (*spe1*), which synthesize the substrates used by Rfs (Fig. 6D).

Under oxidative stress, rhizoferrin production increased by two-folds at any time point when assayed in the presence of H_2_O_2_ compared to that in YNB-0.1% not supplemented with H_2_O_2_. Either in the presence or absence of H_2_O_2_, the accumulation of rhizoferrin stabilized after 12 h of growth (Fig. 6E). These data indicated that H_2_O_2_ stimulates rhizoferrin accumulation in the SS of *M. lusitanicus*.

The SSs obtained from the different strains cultured for 48 h in YPG-2% supplemented with 0.5 mM H_2_O_2_ revealed that the SS from ∆*rfs* did not exhibit toxicity even in the presence of H_2_O_2_ (Fig. 6F) compared to that in the SS obtained under similar conditions from MU636 and R7B. Moreover, MU636 showed a higher toxicity in the presence of H_2_O_2_ than the SS obtained from the strain cultured in YPG-2% (Figs. 6F and 1A). Meanwhile, after its growth in the presence of H_2_O_2_, the SS from MU636 + *rfswt* showed the highest toxicity (Fig. 6F). These data indicate that *rfs* transcription is positively influenced by macrophage interaction and oxidative stress, and this stressor led to an increase in rhizoferrin production and virulence of *M. lusitanicus*.

### Mitochondrial activity depends on Rfs and is needed for virulence

**Figure 7:**
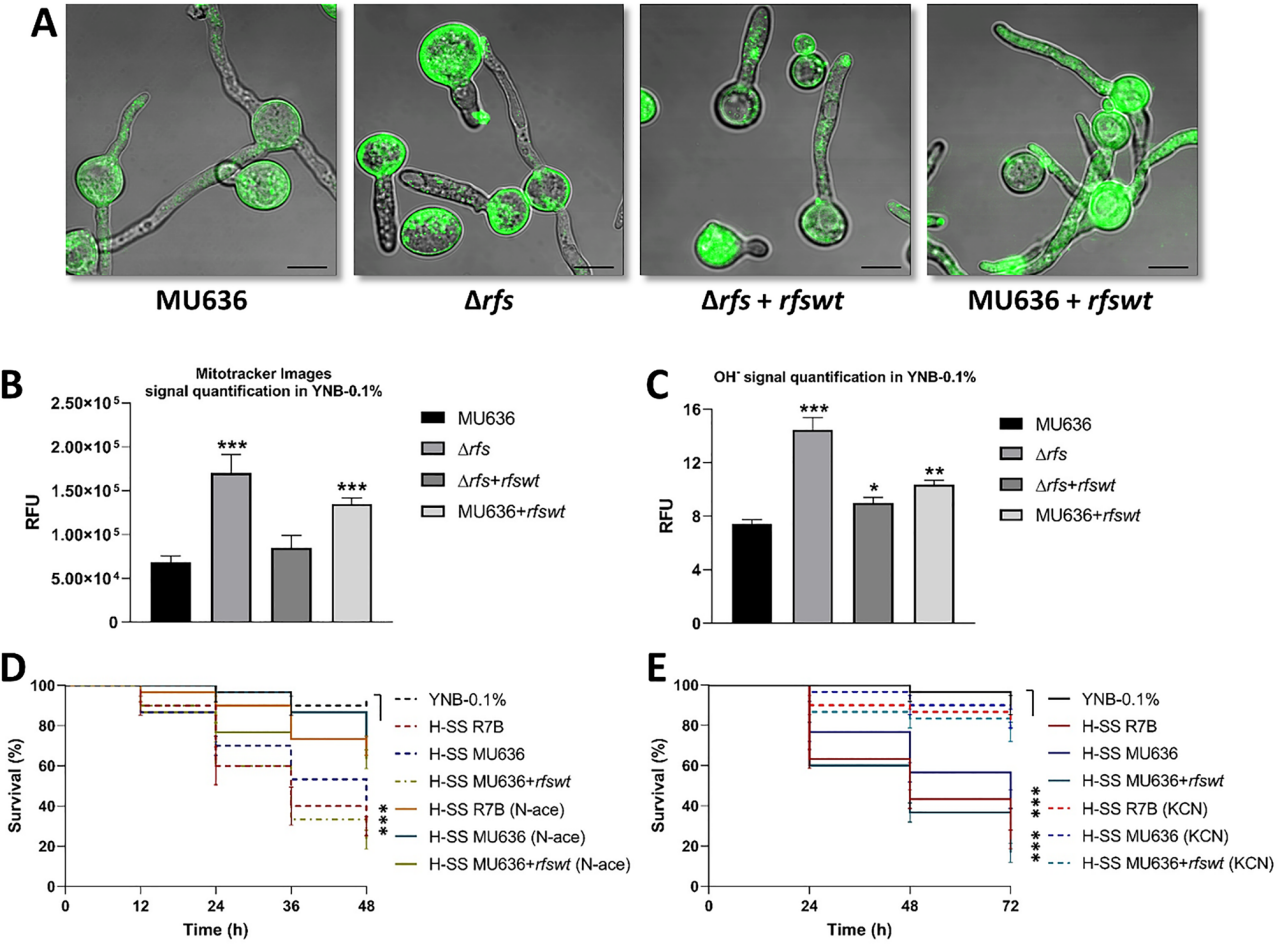
Role of Rfs in the mitochondrial membrane potential, reactive oxygen species generation, and *M. lusitanicus* virulence. Germinules formed at 3 h of growth in YNB with 0.1% glucose (YNB-0.1%) were visualized (**A**) in the presence of MitoTracker Green FM, following which the cells were visualized by confocal microscopy (120 ×), bars = 20 mm. The fluorescence signal was quantified using the (**B**) Mitotracker signal and (**C**) hydroxyl radical (OH^−^) concentration. The results presented are the average of values obtained from four independent experiments. Significance testing was performed using ANOVA with Fisher's exact test, *P < 0.05; **P < 0.01; ***P < 0.001. (**D**) Effect of 10 mM N- acetylcysteine (N-ace) or (**E**) 0.5 mM potassium cyanide (KCN) on the virulence of cell-free supernatants of the culture (SS) from MU636-, R7B-, and *rfs*-overexpression (MU636 + *rfswt*) strains aerobically grown (H-SS) in YNB-0.1%. The SSs were used to treat *Caenorhabditis elegans*. A total of 15–20 nematodes were used per experiment and incubated at 18 °C for 72 h. The results presented are the average of values obtained from four independent experiments. Data were statistically analyzed using the Mantel-Cox test. ***P < 0.01. When results were not considered significant, we did not provide an additional indication (*P* > 0.05).

The results presented suggest that ∆*rfs* or MU636 + *rfswt* could have alterations in oxidative metabolism. To test this hypothesis, we first measured the mitochondrial membrane potential using the fluorescent dye Mitotracker®. ∆*rfs* and MU636 + *rfswt* showed stronger fluorescence signals (≈ 149% and 97%, respectively) than the WT (MU636) or ∆*rfs* + *rfswt* (Fig. 7A,B). ∆*rfs* and MU636 + *rfswt* also produced higher levels of hydroxyl ions (OH^−^) than the WT (Fig. 7C). We also determined whether oxidative metabolism or some of its sub-products, such as reactive oxygen species (ROS), could modulate the virulence of *M. lusitanicus*. To this end, we obtained SSs from strains grown in YNB-0.1% supplemented with either the ROS scavenger N-acetylcysteine (10 mM, N-ace) or sub-inhibitory concentrations of potassium cyanide (0.5 mM, KCN)—an inhibitor of the electron transport chain— that allowed mycelial growth (Fig. S6). The SSs from the WT and MU636 + *rfswt* obtained after incubation with N-ace or KCN increased nematode survival compared to that observed under control conditions (Fig. 7D,E). These data strongly suggest that Rfs is needed for adequate oxidative metabolism and particularly for ROS production, which influences the virulence of *M. lusitanicus*.

### The cAMP-PKA pathway modulates virulence and rhizoferrin accumulation in *M. lusitanicus*

**Figure 8:**
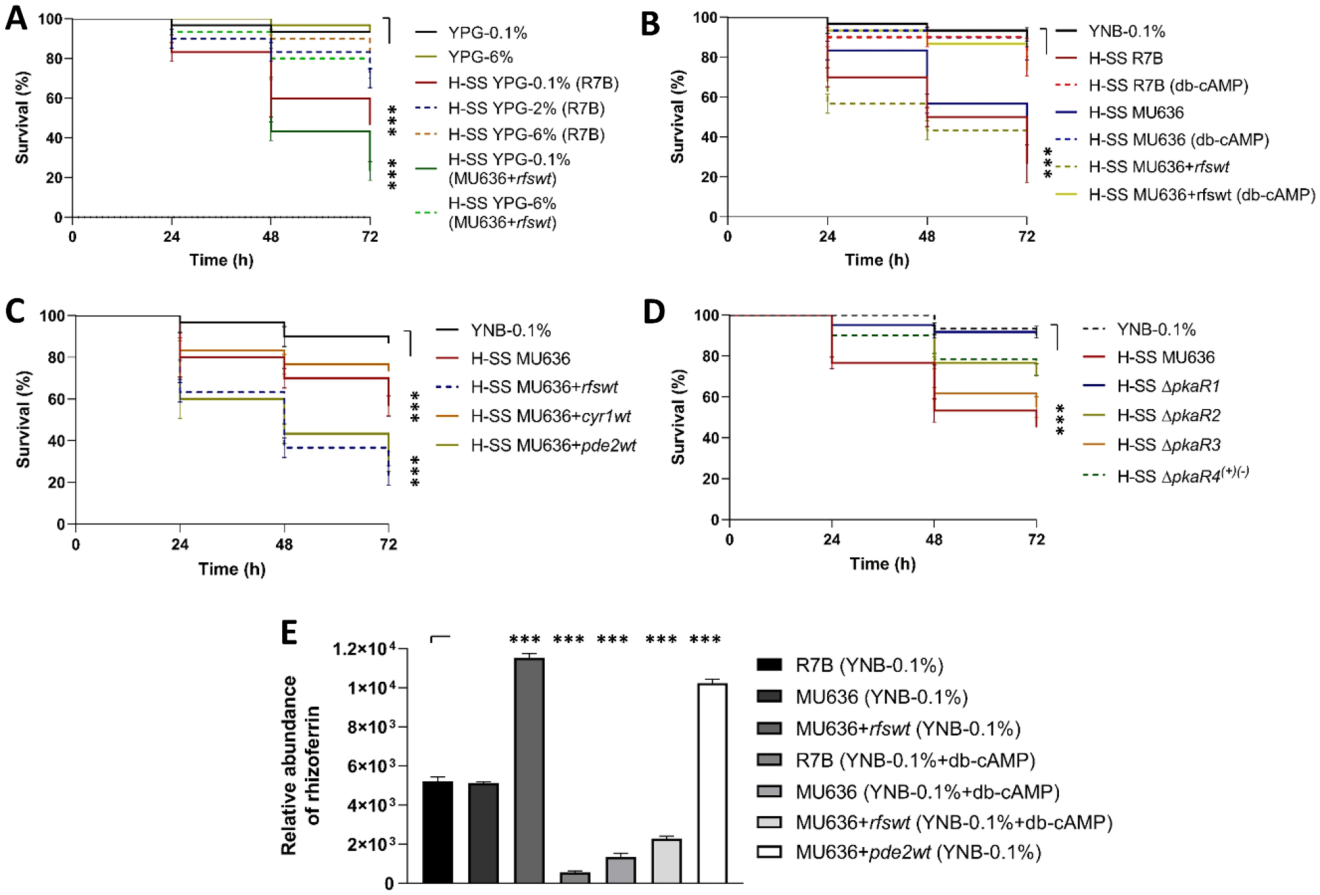
Involvement of the cAMP-PKA pathway in the regulation of *rfs*, accumulation of rhizoferrin, and virulence of *M. lusitanicus*. (**A**) Effect of YPG supplemented with 0.1%, 2%, 4%, and 6% glucose (YPG-0.1%, YPG-2%, YPG-4%, and YPG-6%) on the virulence of the cell-free supernatant of the culture (SS) obtained from R7B- and *rfs*-overexpression (MU636 + *rfswt*) strains. (**B**) Effect of the SSs of MU636, R7B, and *rfs*-overexpression (MU636 + *rfswt*) strains obtained after aerobic growth (H-SS) in YNB containing 0.1% glucose (YNB-0.1%) in the presence or absence of 3 mM dibutryril cAMP (db-cAMP) on *Caenorhabditis elegans*. (**C**) Effect of SSs of adenylyl cyclase 1 (*cyr1*)- and phosphodiesterase 2 (*pde2*)-overexpressing *M. lusitanicus* on *C. elegans*. (**D**) Effect of SSs obtained under the aerobic growth of different *M. lusitanicus* mutants of the regulatory subunit of PKA on *C. elegans* survival. The results presented are the average of the values obtained from four independent experiments. Data were statistically analyzed using the Mantel-Cox test. ***P < 0.01. When results were not considered significant, we did not provide an additional indication (*P* > 0.05). (**E**) Quantification of rhizoferrin in the SSs obtained after the aerobic growth of R7B in YNB-0.1%, MU636, and *rfs*-overexpression (MU636 + *rfswt*) in the absence or presence of 3 mM db-cAMP, or MU636 overexpressing phosphodiesterase 2 (*pde2*). Significance testing was performed using ANOVA with Fisher's exact test, *P < 0.05; **P < 0.01; ***P < 0.001.

Mycelial development, virulence, and oxidative metabolism are modulated by the cAMP-PKA pathway in *M. lusitanicus*^22,33^. Increasing the glucose concentration to 4% and 6% or adding 3 mM dibutiryl-cAMP (db-cAMP) to the YPG medium during the aerobic growth of MU636 and MU636 + *rfswt* decreased the virulence compared to that of strains grown in YPG-0.1% alone (Fig. 8A,B).

Between adenylate cyclase (*cyr1*) and phosphodiesterase (*pde2*) overexpression in the MU636 strain, only *pde2* overexpression increased the toxicity of the SS (to similar levels as that in the SS from MU636 + *rfswt*) compared to that of SS from the WT, whereas *cyr1* overexpression did not affect SS toxicity compared to that of the WT (Fig. 8C). Subsequently, the SSs obtained from different mutants of *pkaR*-encoding subunits (∆*pkaR1*-∆*pkaR4*) undergoing aerobic growth in YNB-0.1% were tested against the nematode. To ensure the same genetic background for all *pkaR* mutant strains, ∆*pkaR2* was generated from MU402 (Fig. S8) instead of R7B^34^. Only the SS obtained from ∆*pkaR1* did not induce the death of nematodes (Fig. 8D). The relative quantification of rhizoferrin production showed that, with the addition of db-cAMP, the rhizoferrin levels in the SS decreased in the WT and MU636 + *rfswt*; by contrast, *pde2* overexpression increased rhizoferrin accumulation in the SS compared to that in the WT (Fig. 8E). This indicates the involvement of the cAMP-PKA pathway in the regulation of virulence through the PkaR1 subunit, which controls *rfs* expression and rhizoferrin production.

## Discussion

The findings of this study revealed the pivotal role of Rfs in *M. lusitanicus* virulence. The virulence assays using *C. elegans* revealed that R7B exhibits greater virulence than MU636 and MU402. This is possible since R7B (*leuA*^*−*^, *pyrG*^+^) was chemically mutagenized to obtain MU402 (*leuA*^*−*^, *pyrG*^*−*^), a strain with a second selective marker (*pyrG*^*−*^, besides *leuA*^*−*^) for *M. lusitanicus.* Besides *pyrG*, more genes could be mutated, resulting in a phenotype with lower virulence than R7B^35^. Meanwhile, MU636 (*leuA*^*-*^, *pyrG*^+^) was derived from MU402, in which a wild-type *pyrG* was inserted into the locus of *pyrG*^36^. However, these strains responded to different stimuli in a similar manner in terms of virulence, *rfs* mRNA accumulation, and rhizoferrin production.

Examination of the SSs revealed that the component responsible for its toxicity was a molecule with as low MW as rhizoferrin. The deletion of *rfs*, which encodes the only enzyme responsible for the synthesis of rhizoferrin^13^, resulted in a complete loss of the production of the molecule and toxicity of SS and low virulence of spores, whereas the rhizoferrin levels and virulence were elevated in MU636 + *rfswt*.

The toxicity of the SS obtained after aerobic growth from WT strains was positively influenced by factors that stimulate oxidative metabolism, such as low glucose concentration or the presence of an inorganic nitrogen source, in the medium. Compared to the presence of peptone, the presence of ammonium sulfate in the *M. lusitanicus* culture induces higher toxicity^37^. Our results indicated that ammonium sulfate increased oxidative metabolism in *M. lusitanicus*. In this context, the increase in glucose concentration in the culture induced a Crabtree-positive effect, stimulating fermentative metabolism, despite the presence of oxygen, in this fungus^15,18^. In some *Mucor* species, oxidative metabolism stimulates hyphal growth^38^, which correlates with an increase in virulence^20^. This explains why the SS obtained after the growth of yeast cells or that obtained from mycelia grown in media with high glucose levels showed lower toxicity. Contrarily, a higher virulence was achieved when a lower concentration of glucose (0.1%) or glycerol was used for fungal growth.

Meanwhile, the toxicity of the SS from *M. lusitanicus* depended on low iron concentrations in the culture, which positively correlated with *rfs* mRNA upregulation. Siderophores are produced under low iron concentrations in the medium^39^, and their production is modulated by carbon or nitrogen sources or chemical or physical factors, such as pH or temperature^40,41^. An *in-silico* analysis of the promoter region of *rfs* revealed the transcriptional element Yap1 (TTACTCA), which is involved in the oxidative stress response^42,43^, and the iron response element SreA (ATCWGATAA)^42,43^, suggesting a regulation by these factors (Table S4). Our findings indicate that an increase in oxidative metabolism increases the production of rhizoferrin and virulence in *M. lusitanicus*. This could be explained in part by the use of citrate (synthesized by citrate synthase, Cit1) and diaminobutane (produced by ornithine decarboxylase, Spe1), both derived directly and indirectly from mitochondrial metabolism^13^, as substrates for rhizoferrin synthesis. *cit1* and *spe1* mRNA levels increased owing to the stimulation of oxidative metabolism.

In *M. lusitanicus*, as has been described in other fungi, siderophore production, available oxygen, and redox balance are closely linked^43^. In support of the connection between oxidative metabolism and the need for mycelial development for the complete virulence of *M. lusitanicus*, mutation in *cnbR*, which encodes the regulatory subunit of calcineurin, induces yeast development, even in the presence of oxygen, and this mutant is less virulent than the WT^19^. It would be interesting to decipher the oxidative-fermentative metabolism in ∆*cnbR* and its correlation with rhizoferrin production.

In agreement with the need for oxidative metabolism and the enhancement of *M. lusitanicus* virulence, the presence of KCN (0.5 mM), an inhibitor of the electron transport chain, during aerobic growth led to a decrease in the toxicity of the corresponding SS from MU636 + *rfswt* and WT. The inhibitor (at 15 mM) induced yeast growth, even in the presence of oxygen, indicating a correlation among non-functional mitochondrial metabolism and higher fermentative metabolism during aerobic growth^38^. Similarly, the presence of N-acetylcysteine, a ROS scavenger, decreased the toxicity of the SS of the fungus, even under *rfs* overexpression. These results suggest that the toxic effect of rhizoferrin in the SS of *M. lusitanicus* requires efficient oxidative metabolism. In agreement with these results, the induction of oxidative stress upon the addition of H_2_O_2_ increased the virulence of wild-type strain of *M. lusitanicus*, with an increase in the mRNA levels of *rfs* and accumulation of rhizoferrin. The production of the bacterial siderophore enterobactin is also stimulated by oxidative stress^44^. The authors proposed the participation of enterobactin in regulation occurring in response to oxidative stress, because *Escherichia coli* strains lacking the enterobactin system are more susceptible to H_2_O_2_ and paraquat^44^. Our study showed similar results, as ∆*rfs* exhibited increased susceptibility of the spores to H_2_O_2_ (Fig. S7). Consistent with these results, an increase in the mitochondrial membrane potential and the levels of the hydroxyl radical (OH^−^) was observed in ∆*rfs* (≈ 90%) or MU636 + *rfswt* (≈ 30%) compared to the values in the WT. In this context, iron homeostasis is critical for not only cell viability, but also iron deficiency, and its excess increased the oxidative stress, which could involve mitochondrial dysfunction^6,43^. It is likely that oxidative protection by rhizoferrin could be attributed to its ability to scavenge iron intracellularly and avoid the toxic effects of free iron, as has been described for other siderophores^45^, although this hypothesis has not yet been explored. The free iron in the intracellular milieu could participate in Haber–Weiss chemistry, catalyzing the formation of OH^−^, leading to an increase in the oxidative stress, thereby contributing to cellular damage and loss of viability^45^.

Mice infected with ∆*rfs* showed a low death rate, lower fungal burden in the liver, lower expression of IL-6, and higher weight gain than did mice infected with WT; similar results were obtained in the tests with *C. elegans* when the nematodes were incubated with spores or SS obtained from ∆*rfs*, indicating the need for a functional Rfs for virulence. Meanwhile, mice infected with MU636 + *rfswt* showed reduced overall health status, as indicated by a ≈ 20% loss in weight in less than 7 days after infection, a ≈ 60% reduction in survival, and the highest fungal burden among mice infected with spores from the other strains. The lower fungal invasiveness of ∆*rfs* could be explained in part by our finding that *rfs* is needed for adequate macrophage phagocytosis (Fig. S7A) and resistance to oxidative stress (Fig. S7B). In agreement with these observations, *Aspergillus fumigatus*^46^ and *Candida albicans*^47^ defective in oxidative stress response were found to be less invasive and virulent.

**Figure 9:**
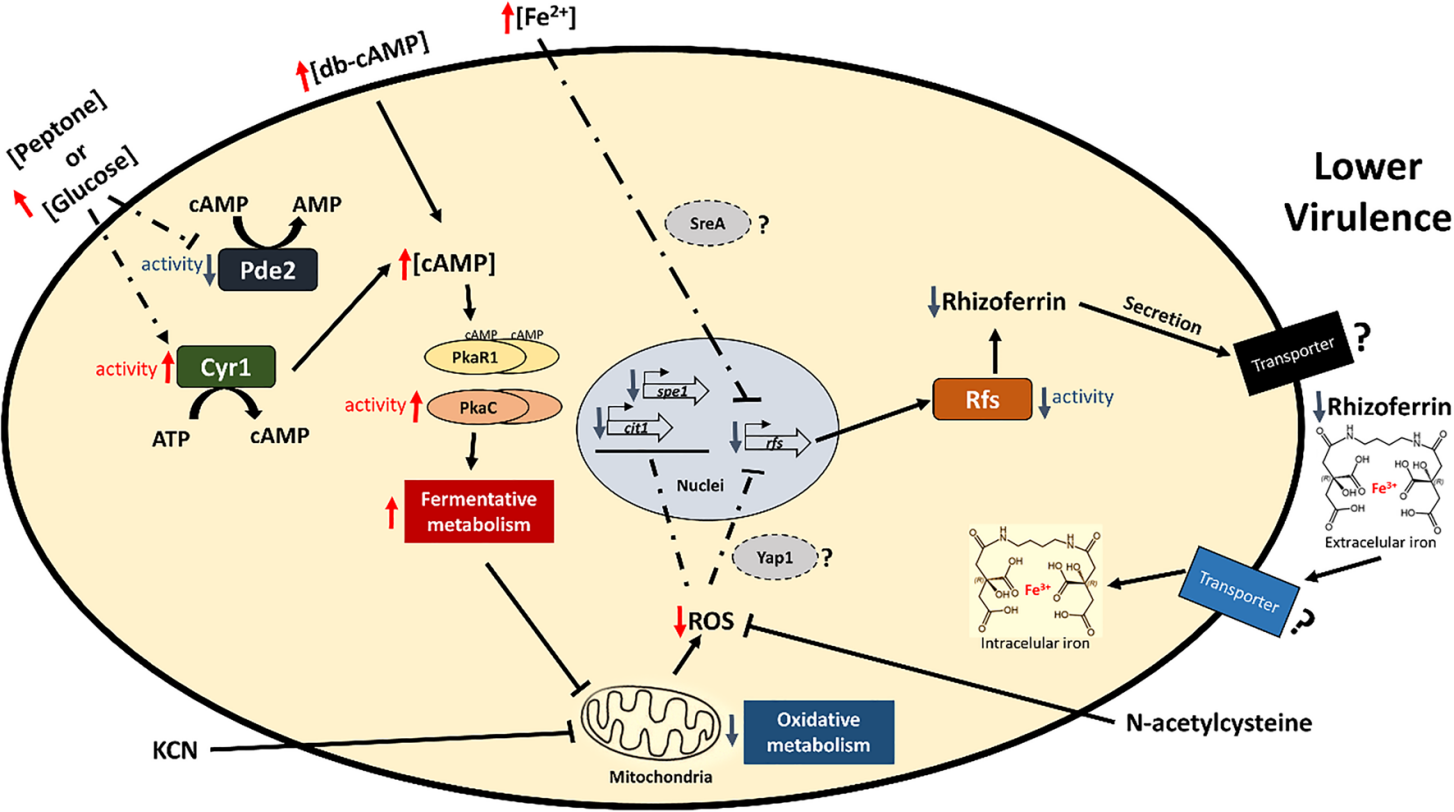
Proposed model for the regulation of rhizoferrin levels via the cAMP-PKA pathway in *M. lusitanicus*. Presence of organic nitrogen (peptone) or high levels of glucose possibly stimulated the enzymatic activity of adenylyl cyclase (Cyr1) and inhibited phosphodiesterase (Pde2), eventually inducing the generation of high levels of cAMP, which binds to PkaR1 (regulatory subunit 1) of PKA (protein kinase A), thereby activating catalytic PKA (PkaC unknown). This stimulates fermentative metabolism and decreases oxidative metabolism, which negatively regulates reactive oxygen species (ROS) production and decreases the mRNA levels of Rfs, probably via the transcriptional regulation of SreA and Yap1. Lower oxidative metabolism correlates with the downregulation of citrate synthase 1 (*cit1*) and ornithine decarboxylase (*spe1*), which synthetize Rfs substrates. This leads to lower levels of rhizoferrin accumulation in the cell exterior, correlating with decreased virulence. In the same context, excess Fe^2+^ led to the transcriptional downregulation of *rfs*, which correlated with lower virulence. Rhizoferrin chelates extracellular iron and transports it to the intracellular space; intracellular rhizoferrin could store iron and help avoid free iron toxicity. Meanwhile, decreased cAMP, low levels of glucose, presence of oxidative stressors, such as H_2_O_2,_ or stimulation of the activity of Pde2 produced the contrary effect, inducing oxidative metabolism and increasing rhizoferrin production, which correlates with increased virulence of *M. lusitanicus*.

The rhizoferrin content in the SS was directly correlated with the virulence of the strains, with MU636 + *rfswt* being the most virulent. The findings of this study suggest that the cAMP-PKA pathway controls the rhizoferrin content (Fig. 9). The activation of the PKA pathway through the mutation of *pkaR1* decreased oxidative metabolism and increased fermentative metabolism and yeast growth in *M. lusitanicus*^22^. SS obtained from ∆*pkaR1* induced the lowest nematode death compared to those obtained from the other *pkaR* mutant strains. Furthermore, the addition of glucose at high levels and db-cAMP during aerobic growth led to lower virulence, even in MU636 + *rfswt,* and a lower rhizoferrin accumulation in the SS. *pde2* overexpression increased the toxicity of the SS and rhizoferrin accumulation. However, we did not observe a lower toxicity than that in the WT when *cyr1* was overexpressed; it is likely that even under *cyr1* overexpression, the endogenous cAMP levels are tightly regulated, or another adenylyl cyclase-encoding gene could be participating in the regulation of cAMP synthesis under these conditions. The mRNA levels of *cyr1* and *pde2* are primarily upregulated during yeast and mycelial growth, respectively^48^. The activity of the products of these genes seems to be important for controlling fermentative-oxidative metabolism via the regulation of cAMP levels in *M. lusitanicus*. Some heterotrimeric G subunits and Arf proteins from *M. lusitanicus* have been implicated in virulence, PKA regulation, and control of fermentative-oxidative metabolism^22,25,28^. Further studies are warranted to understand their contribution to the role of rhizoferrin in this fungus.

The cAMP-PKA pathway has been implicated in several biological functions in fungi, including virulence^49,50^. This pathway also controls critical components for iron uptake, such as ferroxidases and permeases, in the dimorphic basidiomycetes *Cryptococcus neoformans*^50,51^ and *Ustilago maydis*^52,53^. *M. lusitanicus* could secrete rhizoferrin to obtain iron from the host, which stimulates its growth, invasiveness, and virulence, as observed in certain bacteria^54^. However, in the context of a free-living organism such as *M. lusitanicus*, rhizoferrin could be physiologically relevant in the resistance of conditions that increase oxidative stress, e.g., the presence of toxic heavy metals. As reported in bacteria, the presence of these siderophores increases tolerance and growth in the presence of heavy metals^55^. This hypothesis could be relevant to subsequent studies on the role of Rfs and rhizoferrin in tolerance against heavy metals, among other stressors that increase oxidative metabolism in *M. lusitanicus*.

In conclusion, the product of *rfs* is important to avoid phagocytosis, increase resistance to H_2_O_2_ and tissue invasiveness, and enhance the virulence of *M. lusitanicus*. The production of the transcript of the Rfs-encoding gene and accumulation of rhizoferrin are regulated by oxidative metabolism. Transcriptional *rfs* regulation and rhizoferrin accumulation depend on the cAMP-PKA pathway. To the best of our knowledge, this is the first report of a fungal siderophore regulated by the cAMP-PKA pathway.

## Methods

### Fungal strain, spore germination, and growth quantification in aerobic and self-anaerobic conditions

*M. lusitanicus* MU402 (*leuA*^*−*^*, pyrG*^*−*^)^35^, a uracil and leucine auxotroph derived from R7B^56^, was subjected to mutation of the *rfs* and *pkaR2* genes; information for the strains is provided in Table S1. Spores were obtained or germinated in different culture media, as described previously^22^. Vogel’s medium (minimal chemically defined medium) was prepared as described^17^ and supplemented or not with 200 μM FeCl_2_ for further quantitation of *rfs* mRNA. Aerobic and anaerobic growth; spore harvesting, counting, and preservation; and germination percentage determination were performed as previously reported^22^.

Culture media were supplemented as needed with N-ace (Sigma-Aldrich), H_2_O_2_ (Sigma-Aldrich), KCN (Sigma-Aldrich), and dibutyryl-cAMP (db-cAMP) (Sigma-Aldrich). The biomass and SS obtained after the growth of the fungus under the different treatment conditions were recovered by filtration using 0.2 μm Millipore filters and used for subsequent assays.

### Nucleic acid isolation and quantitative reverse transcription polymerase chain reaction (RT-qPCR)

Samples from *M. lusitanicus*, mouse tissues, and macrophages were used to isolate total RNA and genomic DNA employing an RNAeasy mini kit and a QIAamp DNA Mini Kit, respectively (Qiagen, Venlo, Netherlands), as described previously^22^.

Primers and hydrolysis probes for *tfc-1* (used as a reference housekeeping gene)*, atp9*, *adh1*, and *pkaR1* have been reported previously in RT-qPCR assays for *M. lusitanicus*^25,57^. Primers and hydrolysis probes for *rfs*, *pkaR2*, citrate synthase gene (*cit1*), and ornithine decarboxylase gene (*spe1*) (Table S2) were designed using Biosearch Technologies software (www.biosearchtech.com) to ensure specificity during RT-qPCR assays. *rfs*, *cit1*, and *spe1* sequences were retrieved from the genome database of *M. lusitanicus*^58^. We also analyzed the expression of *IL-6* and *Actb* from *Mus* musculus^20^. RT-qPCR was performed as described previously^57^.

### Targeted *rfs* and *pkaR2* deletion

The 1-kb sequences from the 5' upstream and 3' downstream regions of *rfs* or *pkaR2* were fused to the *pyrG* selection gene using an overlap-PCR approach. During this process, three PCR fragments containing overlapping sequences between *rfs* or *pkaR2* and *pyrG* were PCR-amplified using the following primers (Table S3): rfs-pUFwd, rfs-pURev-pyrG, pkaR2-pUFwd, and pkaR2-pURev-pyrG, which amplified the 1-kb sequence from the 5' upstream region of the start translation codon of *rfs* or *pkaR2*; the oligonucleotides rfs-pDRev, rfs-pDFwd-pyrG, pkaR2-pDRev, and pkaR2-pDFwd-pyrG amplified the 1-kb sequence from the 3' downstream region of the stop translation codon of *rfs* or *pkaR2*. For the oligonucleotide pURev-pyrG, a nucleotide sequence was added at the 3' end and hybridized to the 5' end of *pyrG*, and for the oligonucleotide pDFwd-pyrG, a nucleotide sequence was added at the 5' end and hybridized to the 3' end of *pyrG*. *pyrG* was PCR-amplified from plasmid pMAT1700^59^ using the oligonucleotides PyrG-FWR and PyrG-REV. Three individual PCRs were performed to obtain the DNA fragments from the 5´ region, *pyrG* (2 kb), and the 3´ region, which were purified using agarose gel electrophoresis. Overlapping-PCR was performed using 100 ng of each template at a molar ratio of 1:2:1, each of the oligonucleotides pUFwd and pDRev at 10 μM, and 1 μL of Herculase II Fusion Enzyme (Agilent Technologies, Santa Clara, CA, USA) to generate the replacement fragment for *rfs* or *pkaR2*.

Protoplasts obtained from MU402 were transformed with the replacement DNA fragment (3 μg/transformation), and transformants were obtained as described previously^60^.

### Molecular identification of *rfs* or *pkaR2* mutant strains

Transformants with homologous recombination of the *rfs* or *pkaR2* with the *pyrG* marker were identified by PCR. First, the oligonucleotide 5' CR (forward), which hybridizes to the external region of the 5 ´ end of the recombination fragment, was used to delete *rfs* or *pkaR2* and pyrG-REV (reverse), which hybridizes to the internal region of the selection marker sequence (Table S3). A positive integration event in the locus of *rfs* was determined by PCR amplification of a ≈ 3-kb region in the mutant strains and PCR control of the amplification of the 5′ region of *rfs* or *pkaR2* (≈ 1-kb band). Second, the oligonucleotides 5′ CR (forward) and 3’ CR (reverse), which hybridize outside the 5′ and 3′ ends of the recombination fragment, were used to delete *rfs* or *pkaR2,* allowing amplification of the whole locus of *rfs* or *pkaR2*. A positive recombination event was determined by PCR amplification of the 4.2-kb band for the *rfs* mutant, a 5.3-kb band for the WT genotype, the 4.6-kb band for the *pkaR2* mutant, and a 4-kb band for the WT genotype.

### Construction of Δ*rfs* complemented with the *rfs* WT and overexpression in strains

The complemented Δ*rfs* + *rfswt-* and *rfs*-overexpressing MU636 + *rfswt*, *cyr1*-overexpressing MU636 + *cyr1wt*, and *pde2*-overexpressing MU636 + *pde2wt* strains were obtained by transforming Δ*rfs* and MU636 with the WT alleles cloned in the expression vector pEUKA4^61^. The whole ORFs of *rfs*, *cyr1*, and *pde2* were amplified using the oligonucleotides listed in Table S3 and cloned into the pEUKA4 plasmid. Protoplasts obtained from the germlings of the Δ*rfs* and MU636 were transformed with the pEUKA4 recombinant vector, and the transformants were selected on YNB plates lacking uracil and leucine.

### Quantification of mitochondrial membrane potential and the reactive oxygen species OH^−^

Spores were grown in YNB-0.1% glucose for 3 h. The assay with MitoTracker Green FM (Invitrogen, MA, USA) was performed as previously reported^25^. For the quantification of ROS (OH^−^), we used 3'-(p-Aminophenyl) fluorescein (APF) (Thermo Scientific). Germinating spores were recovered by centrifugation at 2,000 rpm for 5 min and washed three times with HEPES (10 mM, pH 7). Next, spores were incubated for 30 min with 10 μM APF (dissolved in 0.1% dimethylformamide) in HEPES with 110 mM glucose. The treated spores were washed three times with HEPES and resuspended in HEPES with 110 mM glucose. The fluorescence of the samples was quantified at a wavelength specific to APF using a VARIOSKAN LUX (Thermo Scientific) with a 520 nm emission filter and 490 nm excitation line.

### Relative quantitation of rhizoferrin

A total of 800 µL of each SS obtained in YNB-0.1% was added to 200 µL of acetonitrile; the mixture was cleaned in a solid phase extraction cartridge (Supelco select HLB) pre-activated with acetonitrile and equilibrated with 20% (v/v) acetonitrile. The extracts were filtered using a PTFE 0.22 µm membrane and stored at − 20 °C until chromatographic analysis.

Rhizoferrin analysis was conducted by UPLC-ESI-TOF-MS using an ACQUITY UPLC I-Class system (Waters, Singapore) coupled with a Synapt G2-Si mass spectrometer (Waters, UK) equipped with an ESI source, controlled by the MassLynx 4.1 software (Waters, UK). Chromatographic separation was performed using a Luna Omega C18 column (150 mm × 2.1 mm and 1.6 µm; Phenomenex) maintained at 40 °C. Five microliters of each pre-treated sample was injected into the chromatographic system and eluted with the mobile phases: 0.1% aqueous formic acid (A) and acetonitrile (B). Separation was performed at a total flow rate of 150 μL/min using the following gradient program: 0 min, 10% B and 0–5 min, 20% B. The mass spectrometer was operated in ESI-positive mode using the following parameters: capillary voltage, 3,000 V; cone voltage, 30 V; source temperature, 120 °C; desolvation gas temperature, 350 °C; desolvation gas flow, 800 L/h; acquisition mass range, 50–1000 m*/z*; scan time, 0.4 s; data format, centroid. Rhizoferrin was monitored at *m/z* = 437.141 ± 0.01 Da.

### Nematode virulence assay

The virulence assay using the nematode *C. elegans* Bristol N2^62^ was performed as described previously^27^. One milliliter of the SSs or 1000 cells corresponding to hyphae or yeast were obtained after 3 h of aerobic culture or 8 h of anaerobic culture, respectively, and used to inoculate the nematodes.

### Mouse-killing assays

The virulence of spores was assessed in mice as described previously^23,28^, with minor modifications. Briefly, male BALB/c mice (12–16 weeks old, weighing ~ 20 g) were treated with streptozotocin (200 mg/kg; Sigma-Aldrich) to induce a diabetic state (≥ 300 mg/dL glucose in blood serum). Each group of mice (n = 10 mice each) was inoculated intraperitoneally with 3 × 10^7^ spores/animal. Mouse survival and weight were monitored daily after fungal infection for 15 days.

### Ethic statement

To assure the welfare of animals and the ethics of procedures related to animal experimentation, the mouse virulence model protocol was conducted according to the recommendations of the Mexican Federal Regulations for the Use and Care of Laboratory Animals^63^.

Animal care procedures were supervised and approved by the internal biosecurity and bioethics committee of the Instituto de Investigaciones Químico Biológicas de la Universidad Michoacana de San Nicolás de Hidalgo (trade number 06-13/2016). Additionally, this study adheres to standards articulated in the ARRIVE guidelines.

### Macrophage spore killing assay

Mouse RAW267.4 monocytes/macrophages (TIB-71) were purchased from the American Type Culture Collection (Manassas, VA, USA). Cells were maintained and co-cultured with *M. lusitanicus*, as described before^22,28^.

### Image analysis

An Olympus CKX41 microscope equipped with a 40 × objective lens and a DMC-T25 camera (Panasonic, Kadoma, Japan) was used to capture images of spores or spore germination.

### Statistical analysis

Data were evaluated by analysis of variance (ANOVA) and unpaired.

Student’s *t*-test (*P < 0.05; **P < 0.01; ***P < 0.001). Fisher's exact test was used for analysis. The Mantel-Cox test was used for survival analyses. ***P < 0.01. When results were not considered significant, we did not provide an additional indication (*P* > 0.05).

## Supplementary Information

Supplementary Information.

## Data Availability

All data generated in this study are available from the corresponding author, upon reasonable request.
