# Supplementary material for: Secretion of the siderophore rhizoferrin is regulated by the cAMP-PKA pathway and is involved in the virulence of Mucor lusitanicus
Source: Sci Rep. 2022 Jun 23;12:10649. doi: 10.1038/s41598-022-14515-0 (PMC9226013; doi:10.1038/s41598-022-14515-0)
Supplement: Supplementary file 1 — Supplementary Information. [file 41598_2022_14515_MOESM1_ESM.docx]

**Table S1. *Mucor lusitanicus* strains used in this study**

| **Strain** | **Genotype** | **Parental strain/Description** | **Source** |
| --- | --- | --- | --- |
| NRRL3631 | WT | *M. lusitanicus* (sex +)/Used as an avirulent strain | (32) |
| R7B | (*leuA^-^*) | *M. lusitanicus* CBS 277.49 (sex -)/Parental strain of MU402, with phenotype more virulent compared to MU402 and MU636 strains | (10, 57) |
| MU402 | (*leuA^-^, pyrG^-^*) | R7B/Strain used for gene deletions such as *rfs* | (35) |
| MU636 | (*leuA^-^, pyrG^+^*) | MU402/Strain derivated from MU402 with insertion in the locus of *pyrG* with the wild type *pyrG* gene and it has been used to compare similar genetic background with MU402 after gene deletion when *pyrG* is used as selective marker. | (36) |
| Δ*rfs* | (*rfs^-^*, *leuA*^-^ *pyrG^+^*) | MU402/deletion of *rfs* | This work |
| Δ*rfs+rfswt* | (*rfs^+^*, *leuA*^+^ *pyrG^+^*); *rfs* is expressed in pEUKA4 | Δ*rfs* | This work |
| MU636+*rfswt* | (*rfs^+^*, *leuA*^+^ *pyrG^+^*); *rfs* is overexpressed in pEUKA4 | MU636 | This work |
| Δ*pkaR1* | (*pkaR1^-^*, *leuA*^-^ *pyrG^+^*) | MU402 | (33) |
| Δ*pkaR2* | (*pkaR2^-^*, *leuA*^-^ *pyrG^+^*) | MU402 | This work |
| Δ*pkaR3* | (*pkaR3^-^*, *leuA*^-^ *pyrG^+^*) | MU402 | (34) |
| Δ*pkaR4^(+)(-)^* | (*pkaR4^-^*, *leuA*^-^ *pyrG^+^*) | MU402 | (34) |
| MU636+*cyr1wt* | (*leuA*^+^ *pyrG^+^*); *cyr1* is overexpressed in pEUKA4 | MU636 | This work |
| MU636+*pde2wt* | (*leuA*^+^ *pyrG^+^*); *pde2* is overexpressed in pEUKA4 | MU636 | This work |

**Table S2.** **Oligonucleotides and hydrolysis probes for qRT-PCR**

| **Gen**  **(ID number*)** | **Forward oligonucleotide (5´-3´)**  **Reverse oligonucleotide (5´-3´)**  **Probe (5´-3´) FAM-BHQ1** | **Tm (°C) / GC (%)** | **Amplification efficiency (%)** | **Amplicon size (bp)** |
| --- | --- | --- | --- | --- |
| *rfs*  (168439) | CTGGCGAATTAAAGGGCTTTGTC  GGACTGCTTGAGGGTCTCATTG  TCGTGATTTCGGTGGCGTCAA | 65.04/47.83  65.74/54.55  67.72/52.38 | 97 | 74 |
| *pkaR2*  (1427857) | CCGTGCTGCCACTGTGATTG  AAATGCCTTCTTGCCAAGTGTA  TCATGGCCGTCTCAAGTGTGC | 66.77/60  63.35/40.91  67.78/57.14 | 98 | 64 |
| *cit1*  (1405305) | GTGCCCTCGGTGTTACATCTC  GCTTCGCTGCTGAAACTCTTG  TTGATCTGGGATCGTGCTCTCGG | 65.68/57.14  64.56/52.38  68.92/56.52 | 96 | 85 |
| *spe1*  (1291766) | GGCCTCCTTTATCCGCTACTC  TTCGGCGTTGTCAAATGTCA  TGCCCAACAGAACGTCAGCCG | 64.58/57.14/  63.64/45  70.26/61.9 | 99 | 64 |

**Table S3. Oligonucleotides used for PCR assays**

| **Name** | **Secuence 5’-3´** | **Tm(°C)/GC%** |
| --- | --- | --- |
| rfs-pUFwd | GGCGTctgcagTTCAATTTAGCGGCGCAGTGCATAG | 77.54/55.56 |
| rfs-pURev-pyrG | **caagtaccaatgctgaggca**AAAGAGGCCGTCTTCCATGCCTTTT | 79.07/48.89 |
| rfs-pDFwd-pyrG | **cgatagcatggccagtgtac**TGCGCGAGATCTCTCTTTTCCTCTTC | 78.76/52.57 |
| rfs-pDRev | GCGATctgcagTAATGACAGCTGGCGGCAAAGACAG | 77.35/55.56 |
| pyrG-FWR | TGCCTCAGCATTGGTACTTG | 62.88/50 |
| pyrG-REV | GTACACTGGCCATGCTATCG | 63.1/55 |
| rfs 5' CR | CAGACTTGTCAAGATCCAGCAGCTCC | 69.95/53.85 |
| rfs 3' CR | TAACGCCGAGCAGGGAATACTGTCA | 70.11/52 |
| rfs XhoI FWR | AGAGActcgagATGCCTGTCCCCTCTACTGA | 74.47/54.84 |
| rfs NotI REV | TAGTTgcggccgcTTAGACGCGAGGTGCTTGTT | 78.59/57.58 |
| pkaR2-pUFwd | GCCGGtctagacAAATCGTTCCCCAAAGAATCGCTGA | 75.55/51.35 |
| pkaR2-pURev-pyrG | **caagtaccaatgctgaggca**GAACAACAAGCAGAAAGAAGCCGAGA | 78.45/47.83 |
| pkaR2pDFwd-pyrG | **cgatagcatggccagtgtac**TCATAAAGGCATCCTCGCATTACGTG | 78.16/50 |
| pkaR2pDRev | GCCGGtctagaCTTTGCATGTATCATCTCCACACCAC | 75.55/51.35 |
| pkaR2 5' CR | TGACCAAGAGACAACTGCTCACAATGC | 69.86/48.15 |
| pkaR2 3' CR | AACAGCGGCAATCAAAGAATCCCTG | 68.58/48 |
| cyr1 FWR SalI | CCTCGgtcgacATGAATATACCACACGCAAA | 72.5/48.39 |
| cyr1 REV NotI | GTATTgcggccgcTTAGAGTGGCTTGCTTTTAG | 73.95/51.52 |
| pde2 FWR XhoI | CGTAActcgagATGGCGGAACATGACGATGT | 73.81/51.61 |
| pde2 REV NotI | GCAGTgcggccgcCTAAATGTGAATATGGTGTT | 75.56/51.52 |

Lowercase letters represent restriction sites.

The lowercase and bold letters show the region that hybridizes to the *pyrG* gene.

**Table S5. *In silico* analysis of the promoter region of the *rfs* gene**

| **Reported function** | **Transcription factor that repress high-affinity iron uptake, including RIA and SIA during iron sufiency** | **Transcription factor required for oxidative stress tolerance; activated by H_2_O_2_ and mediates resistance to cadmium** |
| --- | --- | --- |
| **TF** | SreA (ATCWGATAA) | Yap1 (TTACTCA) |
| **Position** | -168 F | -154 R |

**
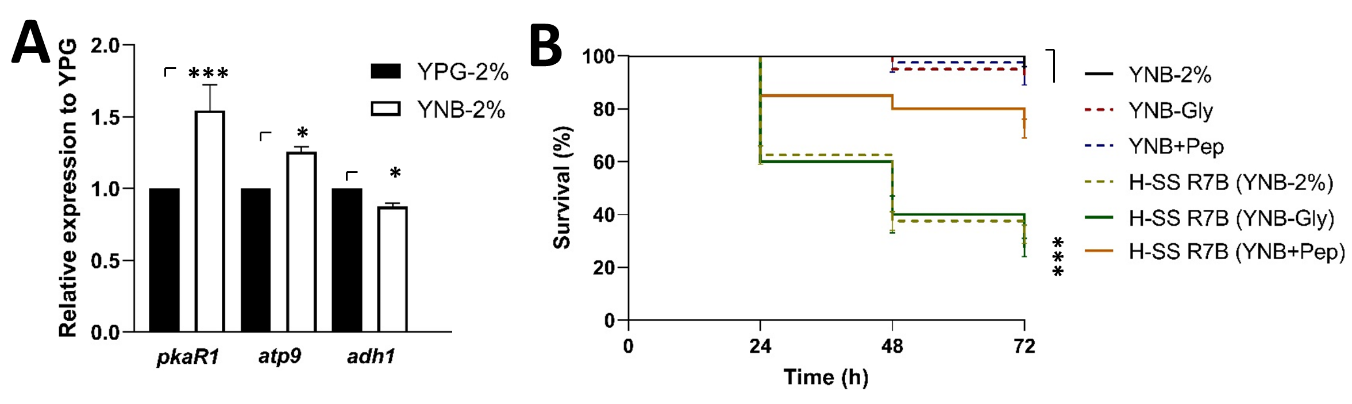
**

**Figure S1. The increase of the oxidative metabolism correlates with the virulence rate of the SS from in *M. lusitanicus*.** The WT strain R7B was grown in aerobic conditions in standard YNB or YPG (both supplemented with 2 %/111 mM of glucose). **A)** The biomass was recovered after 8 h of growth and used to isolate total RNA. RT-qPCR assay was performed to quantify *pkaR1* (mycelial growth marker), *atp9* (mitochondrial activity) or *adh1* (fermentative marker) a ΔΔCt analysis was performed in order to compared the mRNA levels between samples. **B)** Virulence effect against nematodes of the SS obtained from aerobic growth (H-SS) for 48 h in standard YNB (YNB-2%), YNB supplemented with peptone (10 g/L; YNB+Pep), YNB with glycerol (111 mM) as sole carbon source (YNB-Gly). The virulence results presented are the average of four independent experiments. The data were statistically analyzed using unpaired Student’s t test. *, P<0.05; **, P<0.01; ***, P<0.001; and the Mantel-Cox test. ***, *P<*0.01. When results were not considered significant, we did not provide an additional indication (*P>*0.05).


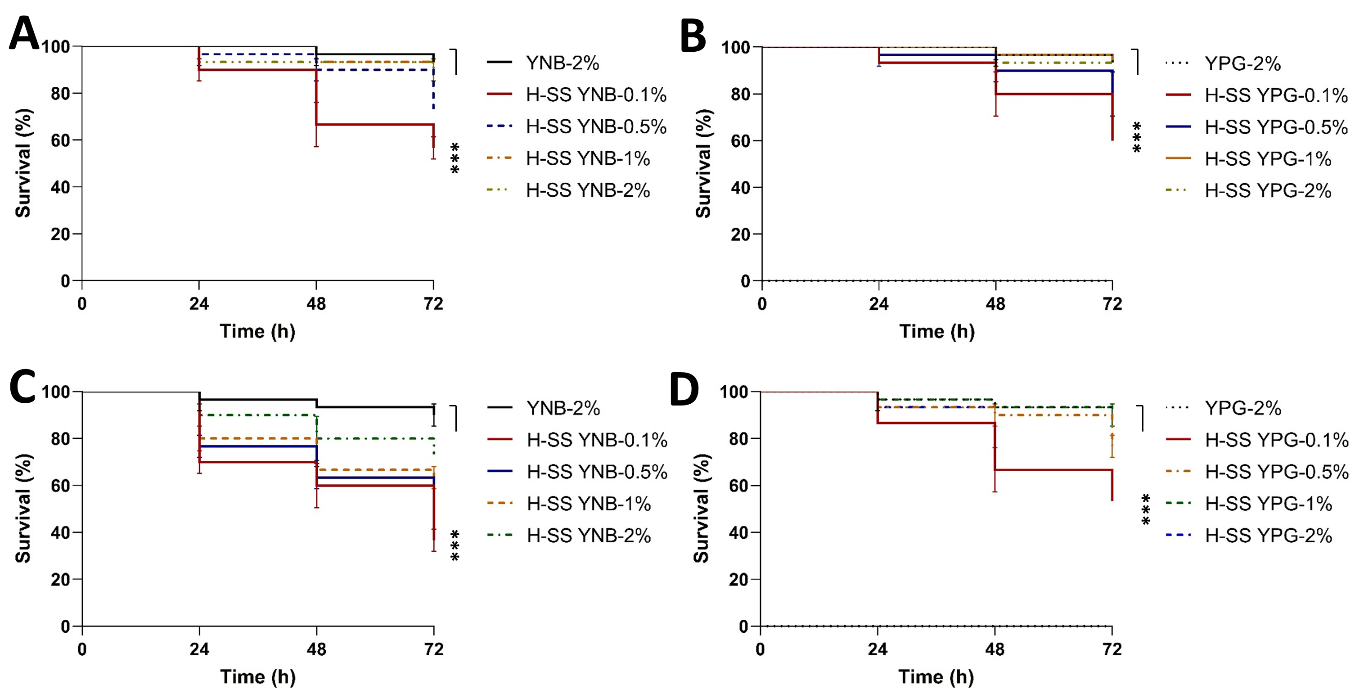


**Figure S2. Concentration of glucose and an inorganic nitrogen source correlates with the virulence rate of the SS from in *M. lusitanicus*.** The WT strains MU636 and MU402 was grown in aerobic conditions (H-SS) using different concentration of glucose in YNB or YPG. Virulence effect against nematodes of the SS obtained from aerobic growth for 48 h was assayed. SS from MU402 in **A)** YNB and **B)** YPG; SS from MU636 in **C)** YNB and **D)** YPG. The virulence results presented are the average of four independent experiments. The data were statistically analyzed using unpaired Mantel-Cox test. ***, P<0.01. When results were not considered significant, we did not provide an additional indication (*P>*0.05).


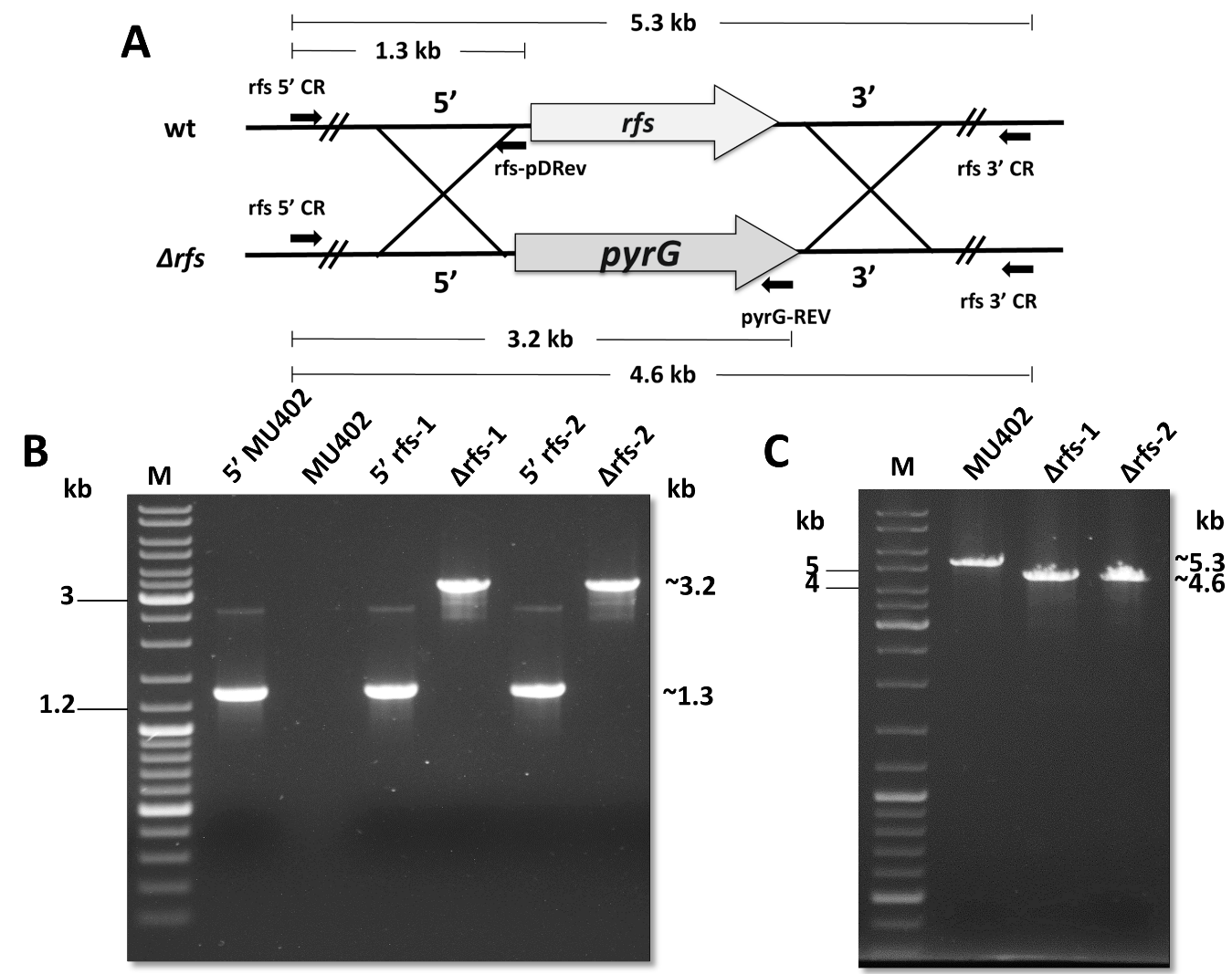


**Figure S3. Deletion of *rfs* gene in *M. lusitanicus*.** **A)** The 5′ and 3′ regions upstream and downstream from the start and stop codons of *rfs*, respectively, were used to flank the *pyrG* selection marker. The diagrams show the recombinant fragment that was used to transform protoplasts of the strain MU402 (*pyrG*−, *leuA*−*)*. **B** and **C**) Molecular confirmation by PCR using specific probes for the 5’ region of *rfs* (1.3 kb for WT-band and 3.2 kb for mutant-band) and the whole locus showed specific recombination event (5.3 kb for WT-band and 4.6 kb for mutant-band).

**Figure S3. Complete gels of panels B and C.**


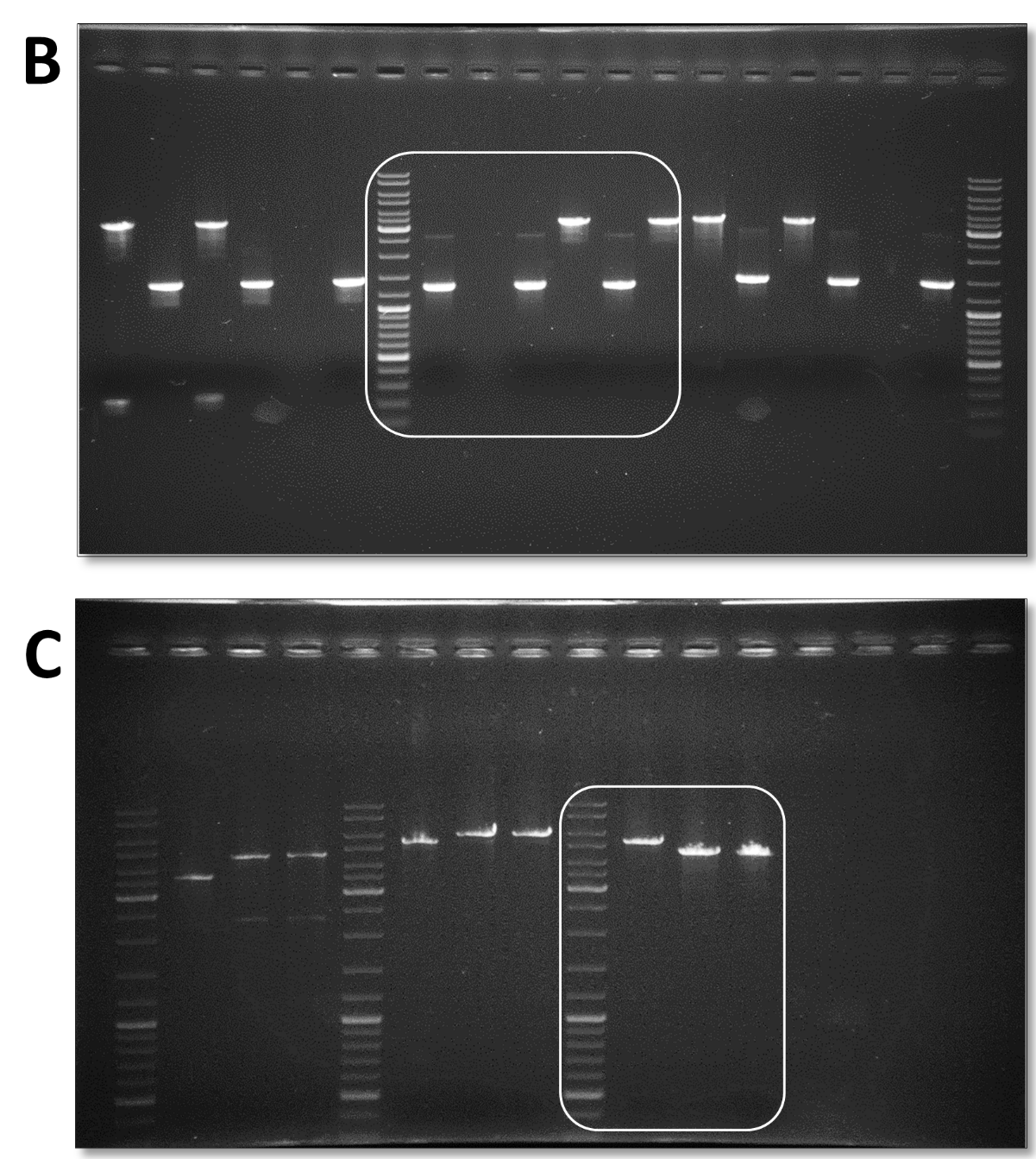


In withe box is shown the corresponding part of the gel that was edited in Fig. S3.


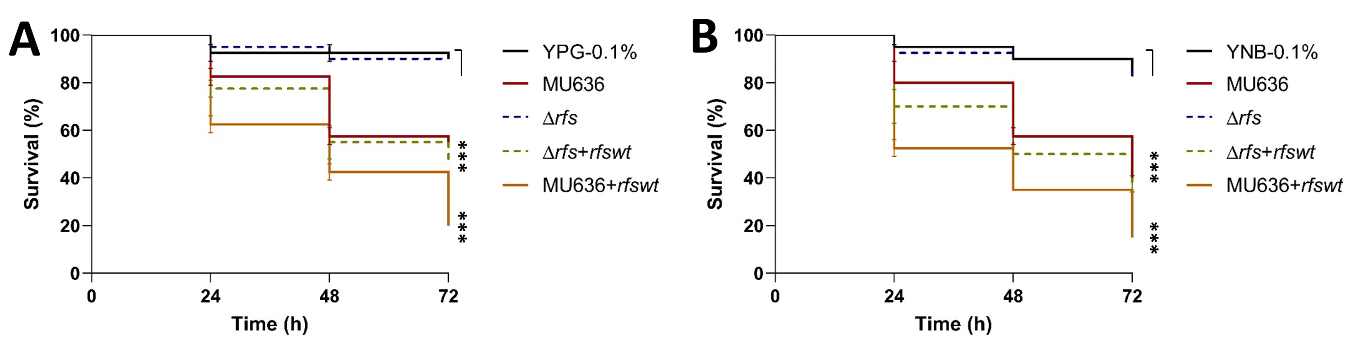


**Figure S4. Virulence rate of spores from *rfs* mutants.** 1000 spores of the different strains were grown in **A)** YPG-0.1% and **B)** YNB-0.1%. Virulence effect against nematodes was assayed. The virulence results presented are the average of four independent experiments. The data were statistically analyzed using the Mantel-Cox test. ***, *P<*0.01. When results were not considered significant, we did not provide an additional indication (*P>*0.05).

**
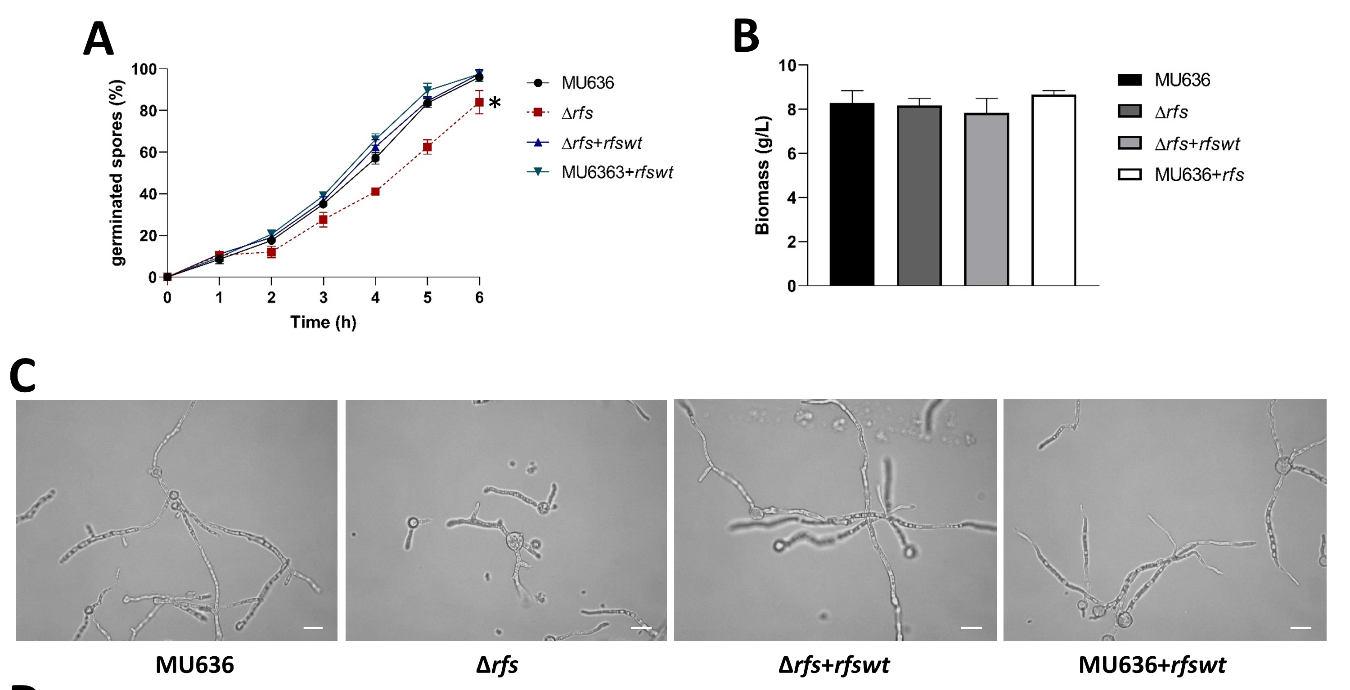
**

**Figure S5. Effect of *rfs* in the aerobic germination of *M. lusitanicus*.** Germination rate was monitored at different time as indicated in **A)** YPG-2% for 6 hours. **B)** The biomass generated after the aerobic growth for 48 h in YPG-2% was registered. **C)** The hyphal morphology was monitored at 4 h in YPG-2%. The bar scale= 20 μm. Significance testing was performed using ANOVA with Fisher's exact test. *, *P<*0.05; **, *P<*0.01; ***, *P<*0.001. When results were not considered significant, we did not provide an additional indication (*P>*0.05).


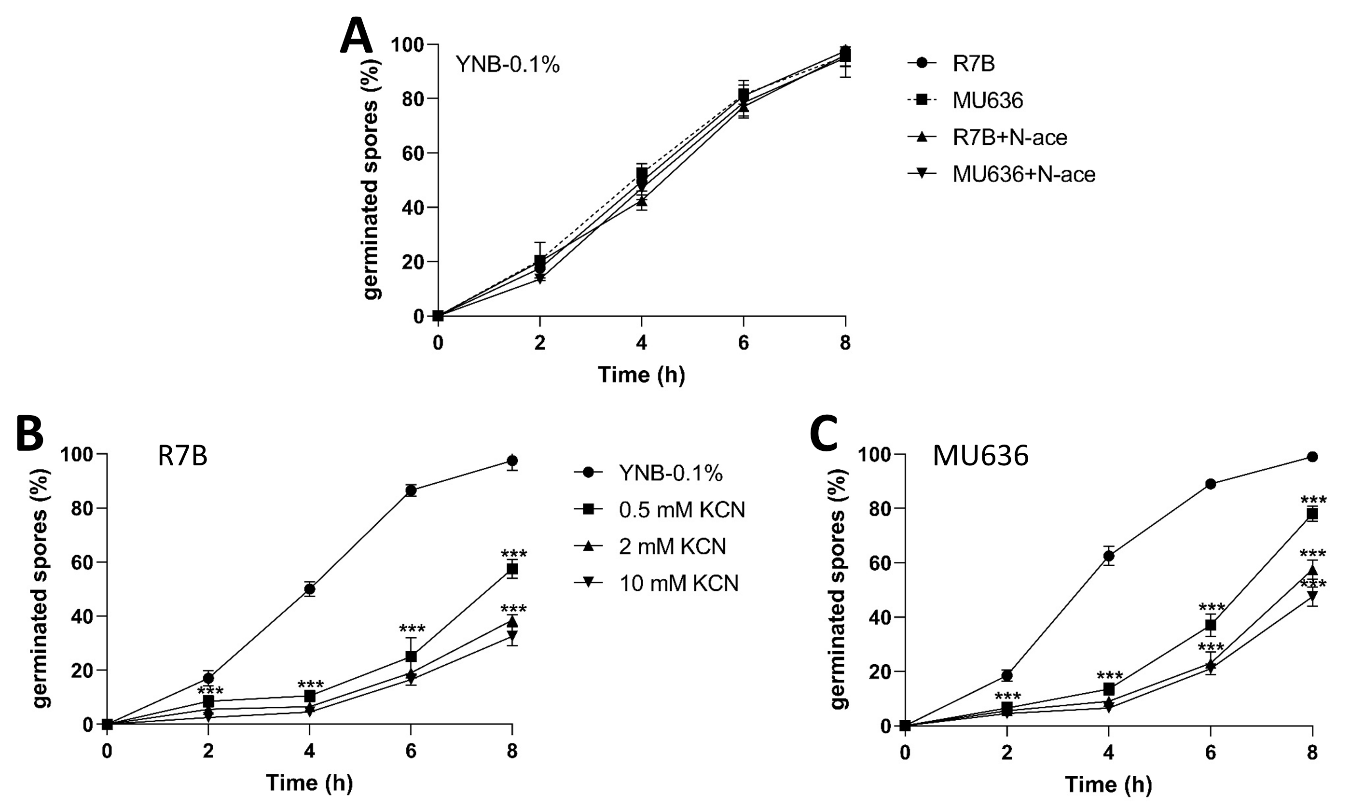


**Figure S6. Effect of N-acetyl cysteine and potassium cyanide in the *M. lusitanicus* aerobic germination.** Germination rate was monitored in YNBB-0.1% medium in **A)** presence of 5 mM N-acetylcysteine (N-ace) or its absence. **B)** R7B and **C)** MU636 strains at different time as indicated in YNB-0.1 % medium supplemented with different concentrations of potassium cyanide (KCN). The hyphal germination was monitored for 8 h The results presented are the average of four independent experiments. Significance testing was performed using ANOVA with Fisher's exact test. The different letters above the lanes denote statistical significance; values with identical letter were not considered significantly different (p<0.05).


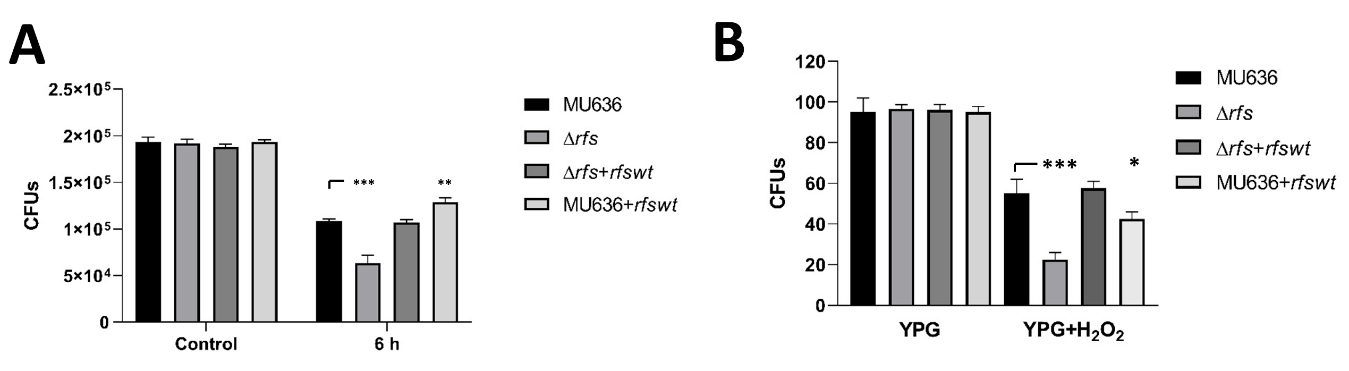


**Figure S7. Macrophage interaction and oxidative stress resistance of *rfs* mutants. A)** The percentage of colony forming units (CFUs) from germinating cells in the presence or absence of macrophages. The results presented are the average of four independent experiments. **B)** Spores from the corresponding strains were incubated with 4 mM of H_2_O_2_ in PBS then the spores were incubated on standard YPG solid medium and the colony formatting units (CFUs) were counted. The results presented are the average of four independent experiments. Significance testing was performed using ANOVA with Fisher's exact test, *, *P<*0.05; **, *P<*0.01; ***, *P<*0.001.

**
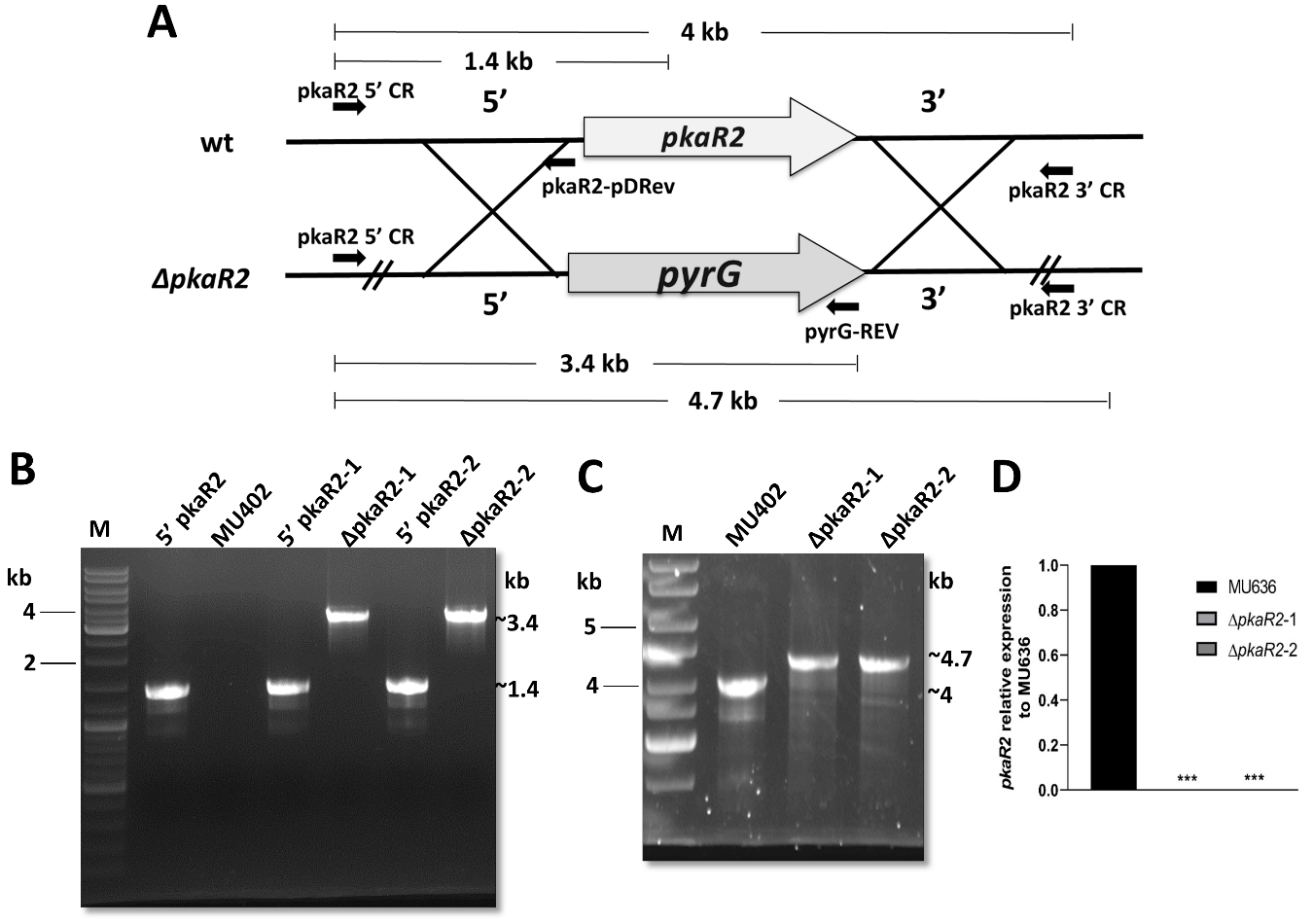
**

**Figure S8. Deletion of *pkaR2* gene in *M. lusitanicus*.** **A)** The 5′ and 3′ regions upstream and downstream from the start and stop codons of *pkaR2*, respectively, were used to flank the *pyrG* selection marker. The diagrams show the recombinant fragments that were used to transform protoplasts of the strain MU402 (*pyrG*−, *leuA*−*)*. **B and C**) Molecular confirmation by PCR using specific probes for the 5’ region of *pkaR2* (1.4 kb for WT-band and 3.4 kb for mutant-band) and the whole locus showed specific recombination event (4 kb for WT-band and 4.7 kb for mutant-band). **D)** RT-qPCR using specific *pkaR2* hydrolysis probe was performed using total RNA from spores of different strains. A ΔΔCt analysis was performed in order to compared the mRNA levels between ΔpkaR2 and WT. The results presented are the average of three independent experiments. Significance testing was performed using ANOVA with Fisher's exact test. *, *P* 0.05; **, *P* 0.01; ***, *P* 0.001. When results were not considered significant, we did not provide an additional indication (*P*> 0.05).

**Figure S8. Complete gels of panels B and C.**

**
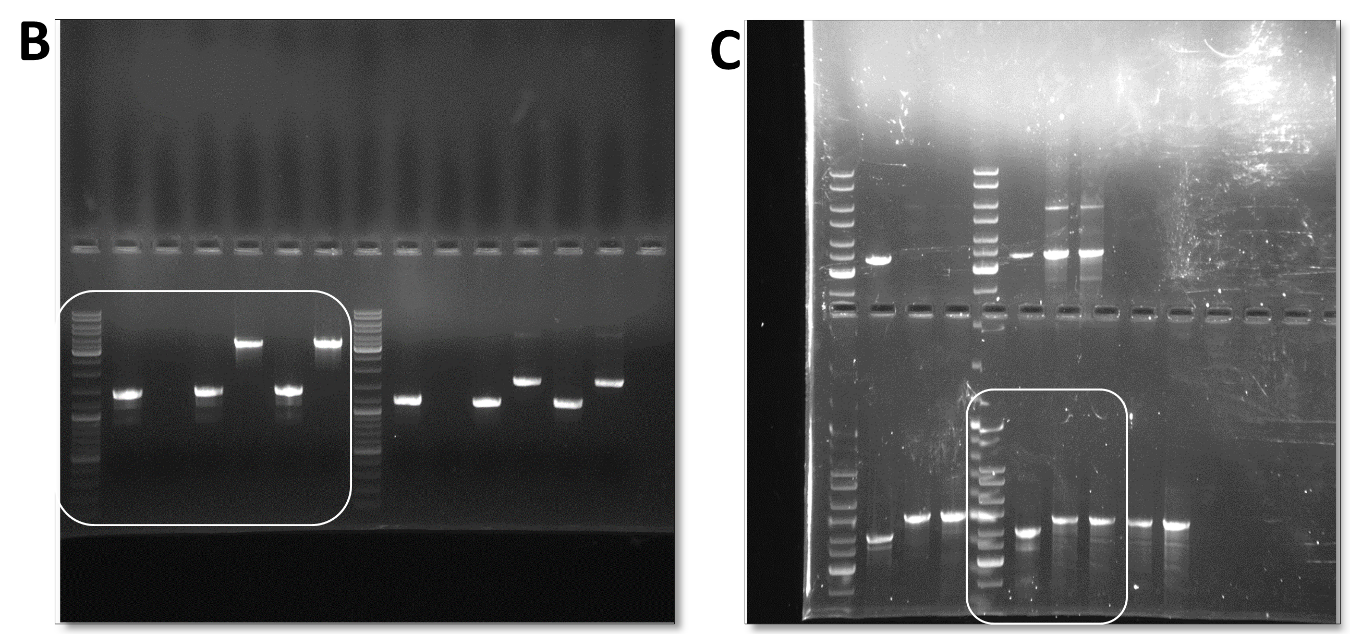
**

In withe box is shown the corresponding part of the gel that was edited in Fig. S8.
